# Copper transporter COPT5 participates in the crosstalk between vacuolar copper and iron pools mobilisation

**DOI:** 10.1038/s41598-018-38005-4

**Published:** 2019-03-15

**Authors:** Àngela Carrió-Seguí, Paco Romero, Catherine Curie, Stéphane Mari, Lola Peñarrubia

**Affiliations:** 1 Departament de Bioquímica i Biologia Molecular and ERI Biotecmed, Universitat de València, Av. Doctor Moliner, 50, ES-46100 Burjassot Valencia, Spain; 2 Laboratoire de Biochimie et Physiologie Moléculaire des Plantes, Institut de Biologie Intégrative des Plantes, Centre National de la Recherche Scientifique (UMR5004), Institut National de la Recherche Agronomique, Université Montpellier II, Ecole Nationale Supérieure d’Agronomie, 34060 Montpellier Cedex 2, France; 3 Present Address: Instituto de Biología Molecular y Celular de Plantas (CSIC-Universidad Politécnica de Valencia), C/Ingeniero Fausto Elio, s/n. 46022 Valencia, Spain; 4 Present Address: Department of Food Biotechnology, Institute of Agrochemistry and Food Technology (IATA-CSIC), Av. Agustin Escardino, 7, 46980 Paterna Valencia, Spain

## Abstract

Copper (Cu) deficiency affects iron (Fe) homeostasis in several plant processes, including the increased Fe requirements due to cuproprotein substitutions for the corresponding Fe counterpart. Loss-of-function mutants from *Arabidopsis thaliana* high affinity copper transporter COPT5 and Fe transporters NATURAL RESISTANCE-ASSOCIATED MACROPHAGE PROTEIN 3/4 (NRAMP3 and NRAMP4) were used to study the interaction between metals internal pools. A physiological characterisation showed that the *copt5* mutant is sensitive to Fe deficiency, and that *nramp3nramp4* mutant growth was severely affected under limiting Cu. By a transcriptomic analysis, we observed that *NRAMP4* expression was highly induced in the *copt5* mutant under Cu deficiency, while *COPT5* was overexpressed in the *nramp3nramp4* mutant. As a result, an enhanced mobilisation of the vacuolar Cu or Fe pools, when the other metal export through the tonoplast is impaired in the mutants, has been postulated. However, metals coming from internal pools are not used to accomplish the increased requirements that derive from metalloprotein substitution under metal deficiencies. Instead, the metal concentrations present in aerial parts of the *copt5* and *nramp3nramp4* mutants conversely show compensated levels of these two metals. Together, our data uncover an interconnection between Cu and Fe vacuolar pools, whose aim is to fulfil interorgan metal translocation.

## Introduction

Transition metals copper (Cu) and iron (Fe) are required by organisms to perform a remarkable wide array of functions that are critical for life. The proteins carrying these metal ions as cofactors mediate diverse biochemical processes, including energy conversion, synthesis and regulation of nucleic acids and lipids, reactive oxygen species (ROS) detoxification, and the signalling events that trigger molecular, cellular and systemic responses^1,2^.

Although the molecular details of metal homeostasis are being deciphered for single metals, the putative cross-interactions among these pathways, which might take place at different levels, remain mostly uncovered. There is considerable experimental evidence to link the Cu and Fe homeostases at different levels^3–5^. Among these potential interactions is metalloprotein substitution such as replacement of *Arabidopsis* Cu/Zn superoxide dismutase (Cu/ZnSOD) with the Fe (FeSOD) counterpart, under Cu scarcity conditions, probably to economise Cu for essential cuproproteins such as plastocyanin^6^. This adjustment is accomplished by the transcription factor PROMOTER BINDING PROTEIN-SQUAMOSA LIKE7 (SPL7)^5,7^ and mediated by *miR398* that regulates *CSD1* and *CSD2* genes that respectively encode cytosolic and chloroplastic Cu/ZnSODs^8^. Conversely, under Fe deficiency, the FeSOD is replaced with the Cu/ZnSOD as Fe down-regulates *miR398*^4^.

Another interaction is metal competition for ligands in long-distance traffic^9^. In both humans and the yeast *Saccharomyces cerevisiae*, Cu is essential for the transport and distribution of Fe due to the participation of Cu-dependent ferroxidases^10,11^. In *Arabidopsis*, the COPT2 plasma membrane protein, a member of the high affinity CTR-like Cu transporter family, denoted COPT, that participates in the entry of reduced Cu in the plasma membrane^12^, has been shown to participate in both Cu and Fe homeostasis^13,14^. COPT5 is another COPT member that localises to the tonoplast and the membrane of the pre-vacuolar/vacuolar compartment in *Arabidopsis* cells^15,16^. *COPT5* is expressed mostly in root vascular tissues and siliques^15^. The *copt5* mutant is sensitive to severe Cu deficiency due to the diminished capacity of *copt5* plants to mediate vacuolar Cu export, which participates in Cu recycling towards the cytoplasm, an especially relevant process under Cu starvation conditions^15^. Furthermore, lack of COPT5 in plants leads to Cu accumulation in roots, and to low Cu in siliques and seeds. This strongly indicates that COPT5 functions in Cu reallocation from roots to reproductive tissues^16^. Accordingly, the *copt5* mutant, which is highly sensitive to cadmium (Cd) toxicity, has lower Cd remobilisation to the aerial parts, which underscores a role for COPT5 in the long-distance translocation of other metals^17^.

Analogously to COPT5, but with Fe instead of Cu, we find transporters NATURAL RESISTANCE-ASSOCIATED MACROPHAGE PROTEIN 3 (NRAMP3) and NRAMP4, which redundantly function in Fe mobilisation from the vacuole, in seeds during germination and in adult plants^18,19^. When grown in Fe-deficient media, the double *nramp3nramp4* mutant is chlorotic and its development is arrested^18^. The mutations in the *VACUOLAR IRON TRANSPORTER 1* (*VIT1*), involved in the influx Fe into the vacuoles during embryogenesis^20^, also compromise the growth of seedlings under Fe-limiting conditions. Recently, a genetic screen showed that the *vit1* mutation suppresses the *nramp3nramp4* phenotype, which illustrates the plasticity of Fe storage in Arabidopsis embryos^21^.

Plants with strategy I, which includes all plants except grasses, acquire Fe after the reduction of Fe^3+^ chelates by a plasma membrane ferric chelate reductase. In *Arabidopsis thaliana*, this reductase is encoded by *FRO2* and the resulting Fe^2+^ is taken up into the cell by the ZIP transporter IRT1. Our current understanding of the Fe deficiency sensor and signalling mechanisms in plants is limited^22,23^. In *Arabidopsis*, the genes involved in the Fe remobilisation and incorporation (e.g. *IRT1* and *FRO2*) are regulated by the helix-loop-helix type transcription factor FIT (bHLH29)^24^. Other bHLH subgroup Ib factors (bHLH38, bHLH39, bHLH100 and bHLH101) are regulated by Fe deficiency^25^ but, while the first two can act in concert with FIT mediating Fe responses^26^, bHLH100 and bHLH101 control Fe homeostasis by a FIT-independent path^27^. Post-transcriptional regulation processes are essential in Fe deficiency responses^28^ and BRUTUS (BTS), which is a functional RING E3 ubiquitin ligase with a hemerythrin domain, has been proposed to function as a potential Fe sensor^29–31^.

Both local and long-distance signals operate in Fe deficiency responses. Members of the Yellow Stripe1-Like (YSL) family, such as YSL1 and YSL3, have been implicated in the transport of multiple micronutrients complexed with nicotianamine (NA) from xylem to parenchyma cells^32,33^. Moreover, the oligopeptide transporter (OPT) family member OPT3 has been involved in the long-distance signalling of Fe status^34–36^. It has been recently suggested that OPT3 might participate in redistributing Fe into phloem when the Fe loading to the parenchyma cells surrounding xylem veins is impaired in the *ysl1ysl3* mutant, which results in proper Fe sufficiency signals being sent to roots^37^.

Fe deficiency is a widespread agronomic and health problem. The results shown herein highlight the effects of the vacuolar Cu pool on Fe homeostasis, which might be considered to obtain crops with optimised nutrient concentrations in edible parts.

## Results

### A genome-wide analysis on *copt5* mutant highlights ion metal mobilisation under copper deficiency

**Figure 1:**
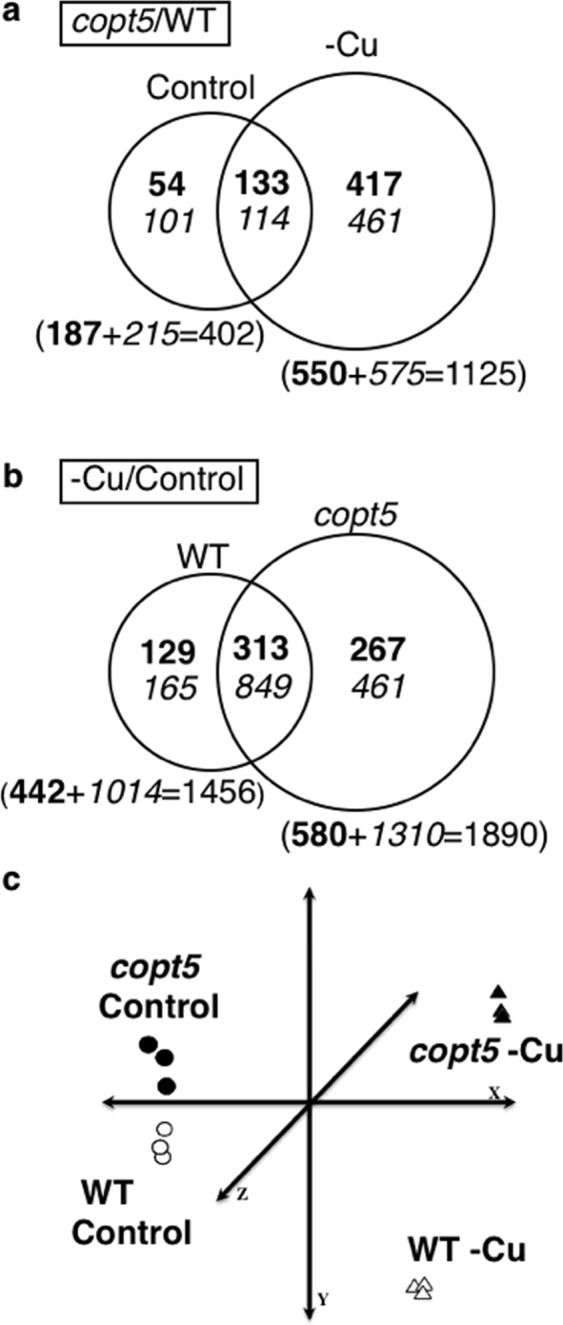
Comparative transcriptomic analyses of the WT and *copt5* mutant seedlings grown under different Cu availability conditions. (**a**) Venn diagrams of the distribution of the differentially expressed genes (DEG) (ANOVA, FDR ≤ 0.01) when comparing *copt5-21* to the WT samples grown under Cu sufficiency (Control) and Cu deficiency (-Cu) media. (**b**) Venn diagrams of the distribution of the DEG when comparing -Cu respect to the Control samples in both the *copt5-2*1 and WT genotypes. The up-regulated (bold) and down-regulated (italics) genes included in these diagrams met a fold-change ≥1.5 cut-off. The numbers in brackets are the sum of all the induced (bold) or repressed (italics) genes under each particular condition. Circle sizes are shown in relation to the total number of DEG for each condition. (**c**) A principal component analysis (PCA), based on the transcriptional profile of the genes that met an ANOVA analysis (*P* ≤ 0.01) from the WT and *copt5*-1 knockout mutant seedlings grown under the Cu sufficiency (Control) and Cu deficiency (-Cu) conditions. The three PCA axes account for 91.37% of the total variance among the genotypes and the Cu availability conditions. Three biological replicates from each condition were used for all the analyses.

To identify the COPT5-dependent functions in *Arabidopsis*, we performed a comparative microarray analysis of 7-day-old *Arabidopsis* wild-type (WT) and *copt5-2* mutant seedlings, grown under severe Cu deficiency and Cu sufficiency conditions. Venn diagrams (Fig. 1a,b) summarise the number of differentially expressed genes (DEG) (ANOVA, FDR ≤ 0.01) in the WT and *copt5-1* in response to these changes in the Cu regime. The most DEG were found in the *copt5-1* mutant when grown under Cu deficiency (Fig. 1a,b). A principal component analysis (PCA) was performed to validate the reproducibility of the microarray data across replications and to cluster samples according to their global gene expression profiles (Fig. 1c). In agreement with the number of DEG shown in the Venn diagrams, the *copt5-1* and WT seedlings grown under optimal control conditions were distributed far from those grown under severe Cu deficiency. In turn, the seedlings from both genotypes grown under severe deficiency were more separated than those grown under the control conditions (Fig. 1c). A set of DEG, described as Cu-responsive genes, was selected to validate the transcriptomic analysis. The comparison made of the fold-change values from the *Arabidopsis* (V4) gene expression, the Agilent technologies microarrays and the expression values obtained by an RT-qPCR analysis run with these genes (multiple linear regression analysis, r^2^ = 0.89), further indicated that the microarray analysis results were robust and reliable (Fig. S1, Table S1).

To further analyse the molecular mechanisms involved in the Cu availability response in which COPT5 played a role, we performed a Gene Ontology (GO) analysis by identifying the biological processes significantly under- or over-represented in the DEG obtained for each comparison considered in the Venn diagrams (Table S2). Many biological processes were commonly regulated in both genotypes in response to severe Cu deficiency. This was the case of the responses to biotic, abiotic and hormonal stimuli, and the toxin and lignin catabolic processes, which suggests that Cu availability regulates primary and secondary metabolisms as well as the responsiveness of the plants to the environment (Table S2). A set of biological processes, which were commonly induced or repressed in both genotypes in response to Cu deficiency, showed that in their regulation was enhanced when COPT5 activity was absent. Among the processes affected in the *copt5-1* mutant, we find responses to reactive oxygen species (ROS) and to ethylene signalling. This fact suggests that COPT5 at least plays a partial role in their regulation. Moreover, the GO analysis also highlighted that the biological processes that were modulated only in the *copt5-1* mutant when grown under Cu deficiency (Table S2), which suggests a primary role for COPT5 in their regulation. This pattern included the cellular carbohydrate, sulphur and glucosinolate metabolic processes, oligopeptide transport, transition metal ion transport and, more specifically, the Fe ion transport.

### Fe-related genes are induced in *copt5* under Cu starvation

In the context of this work, the transition metal ion transport process, induced in *copt5* seedlings in response to Cu deficiency, merits spetial mention. This response might suggest an altered metal and stress response in the *copt5* mutant when Cu is scarce. Therefore, we further investigated the genes included in this process, and found that the genes encoding Cu and Zn transporters and Cu-related proteins (*COPT2*, *HMA2*, *COPT1*, *CCH*, *TCP1* and *ZIP11*), as well as Fe transporters (*OPT3*, *YSL2*, *YSL3* and *NRAMP4*), were induced in *copt5* under Cu deficiency (-Cu) conditions (Table 1). Specifically, these Fe transporters are included in the Fe ion transport biological process and their regulation suggests a role for the COPT5 function in the connection previously reported between Cu and Fe homeostasis^3–5,14^.

**Table 1:** The transition ion metal transporters genes affected in the *copt5* mutant. Differentially expressed genes were identified by applying a false discovery rate (FDR) below 1% and a 1.5-fold change (log_2_ |1.5|).

Locus	Short name	Description	*copt5* + Cu/WT + Cu	*copt5* −Cu/WT −Cu	WT −Cu/WT + Cu	*copt5* −Cu/*copt5* + Cu
AT1G62280	SLAH1	SLAC1 homologue 1	−1.31	1.12	1.55	2.28
AT1G55910	ZIP11	zinc transporter 11 precursor	−1.20	1.18	1.45	2.06
AT5G67330	NRAMP4	natural resistance associated macrophage protein 4	−1.14	1.86	1.27	2.69
AT4G16370	OPT3	oligopeptide transporter	−1.13	2.00	1.26	2.83
AT1G26230	TCP1	TCP-1/cpn60 chaperonin family protein	−1.08	−1.04	1.46	1.51
AT3G56240	CCH	copper chaperone	−1.08	1.25	2.84	3.84
AT3G46900	COPT2	copper transporter 2	−1.05	1.12	3.27	3.85
AT2G32390	GLR3.5	glutamate receptor 3.5	−1.04	1.28	1.44	1.94
AT4G30110	HMA2	heavy metal atpase 2	1.05	1.35	2.16	2.77
AT3G51860	CAX3	cation exchanger 3	1.05	1.51	1.46	2.10
AT5G59030	COPT1	copper transporter 1	1.06	1.36	1.25	1.60
AT5G24380	YLS2	YELLOW STRIPE like 2	1.13	1.23	7.97	8.71
AT5G53550	YSL3	YELLOW STRIPE like 3	1.14	1.37	2.09	2.52

**Figure 2:**
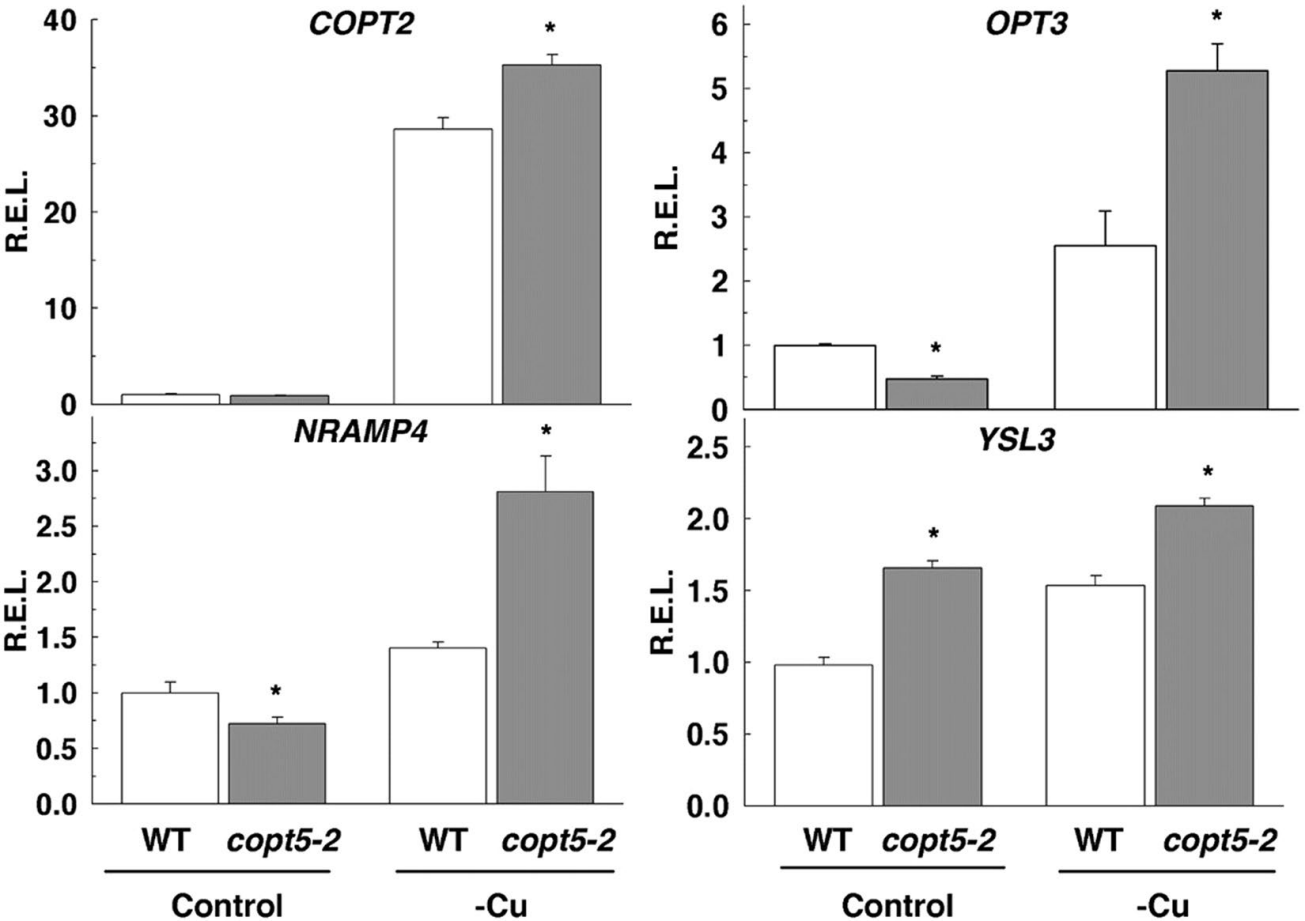
Cu-dependent regulation of the metal-related DEG in the *copt5* mutant. The relative expression levels (R.E.L) of the *COPT2, NRAMP4, OPT3 and YSL3*, genes were determined by qRT-PCR in the 7 day-old WT seedlings grown under Cu sufficiency (Control) and Cu deficiency (-Cu) in the WT (white bars) and *copt5-2* (grey bars). The *mRNA* levels are expressed as relative expression in relation to the WT under control conditions. Bars correspond to arithmetic means (2^−∆∆Ct^) ± standard deviation (SD) (n = 3). For each particular gene, *Indicates statistical differences (P < 0.05) between the values of the WT and *copt5-2* mutant in each condition.

To gain deeper insights into this interaction, the expression analyses of both the representative genes of this biological process and of a set of well-established Fe deficiency related genes was performed by qRT-PCR (Figs. 2, S2 and S3). As expected for a well-known Cu deficiency marker, *COPT2* expression was highly induced under Cu scarcity in both the WT and the *copt5* (Fig. 2). *NRAMP4*, involved in Fe remobilisation from the vacuole^18^, was also up-regulated in the *copt5* mutant compared to the WT under Cu deficiency (Fig. 2). Likewise, *OPT3*, which is involved in the long-distance signalling of the Fe status^34^, and *YSL3*, involved in Fe uptake into the cells^34,38^, were both up-regulated under Cu deficiency in the *copt5* mutant (Fig. 2). We extended these analyses to a set of genes involved in Fe homeostasis, such as the Fe transporters *IRT1* and *YSL1*, Fe reductases *FRO2* and *FRO3*, and transcription factors *FIT*, *BRUTUS* (*BTS*), *bHLH38*, *bHLH39*, *bHLH100* and *bHLH101*. All these genes, except *FIT*, were up-regulated in *copt5* under Cu deficiency compared to the WT seedlings (Figs S2, S3). These results reinforced the idea that Fe homeostasis is altered under low Cu availability in the *copt5* mutant.

### *COPT5* expression depends on Fe availability

**Figure 3:**
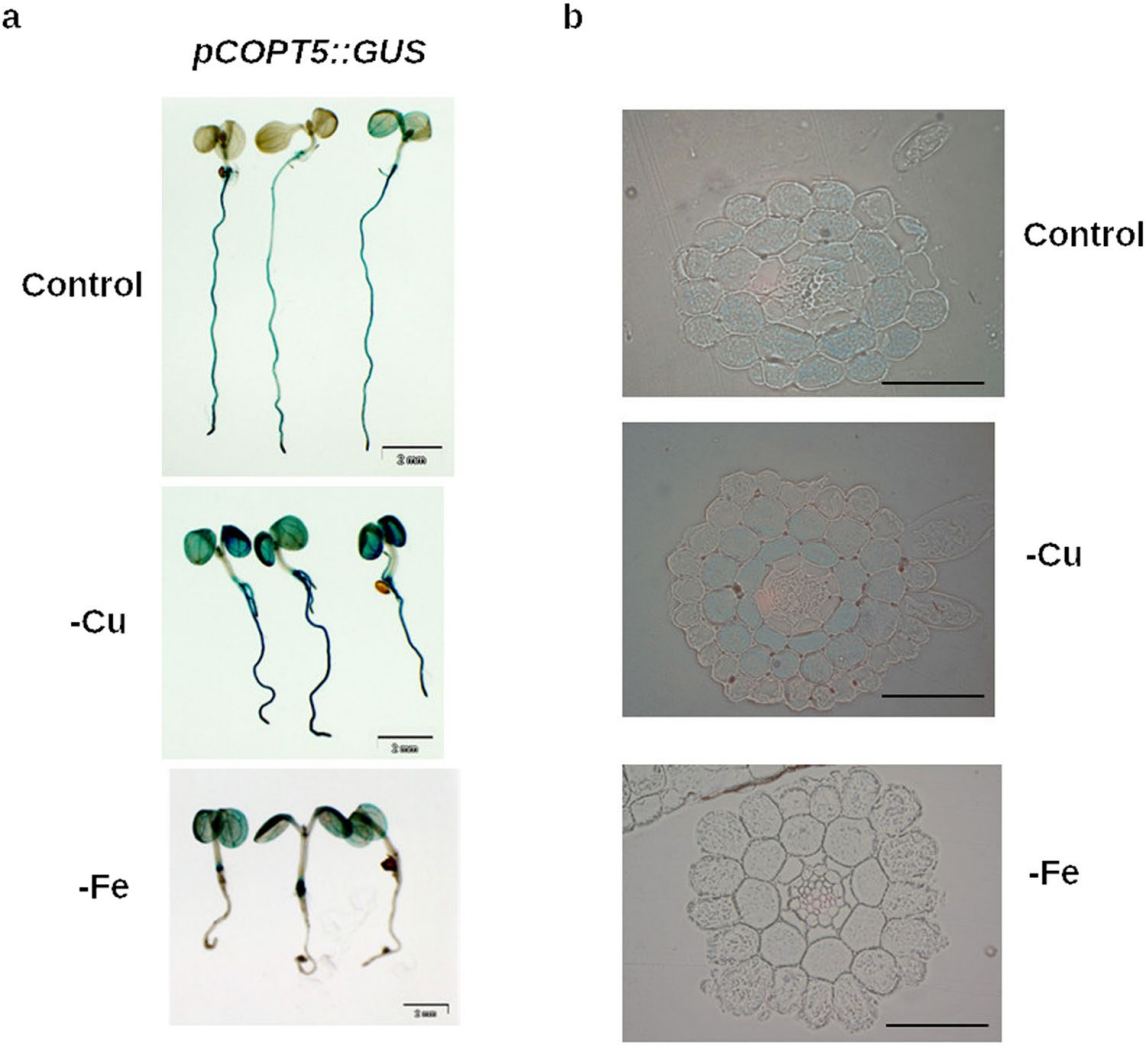
Effect of Fe and Cu deficiencies on *COPT5* gene spatial regulation. (**a**) The *COPT5* expression pattern in the *Arabidopsis pCOPT5::GUS* transgenic plants. GUS staining in three representative 7-day-old seedlings grown under Cu and Fe sufficiency (Control), Cu deficiency (-Cu) and Fe deficiency (-Fe). (**b**) Cross sections of the GUS-stained roots shown in (**a**).

To further address a putative role of COPT5 in Fe homeostasis, *COPT5* expression was checked at different metal conditions (Fig. 3). To this end, 7-day-old *pCOPT5::GUS* transgenic plants were grown under Cu and Fe sufficiency (Control), Cu deficiency (-Cu) and Fe deficiency (-Fe) to show GUS histological activity (Fig. 3a,b). Under metal sufficiency, the *GUS* signal was widely present in roots, especially in the root central cylinder (Fig. 3a). On the contrary, the *COPT5* promoter under Cu deficiency was active mainly in vascular bundles of the cotyledons, cortex and endodermis tissues at the root (Fig. 3b). However, *COPT5* expression disappeared from roots and concentrated in the crown of seedlings grown under Fe deficiency (Fig. 3).

**Figure 4:**
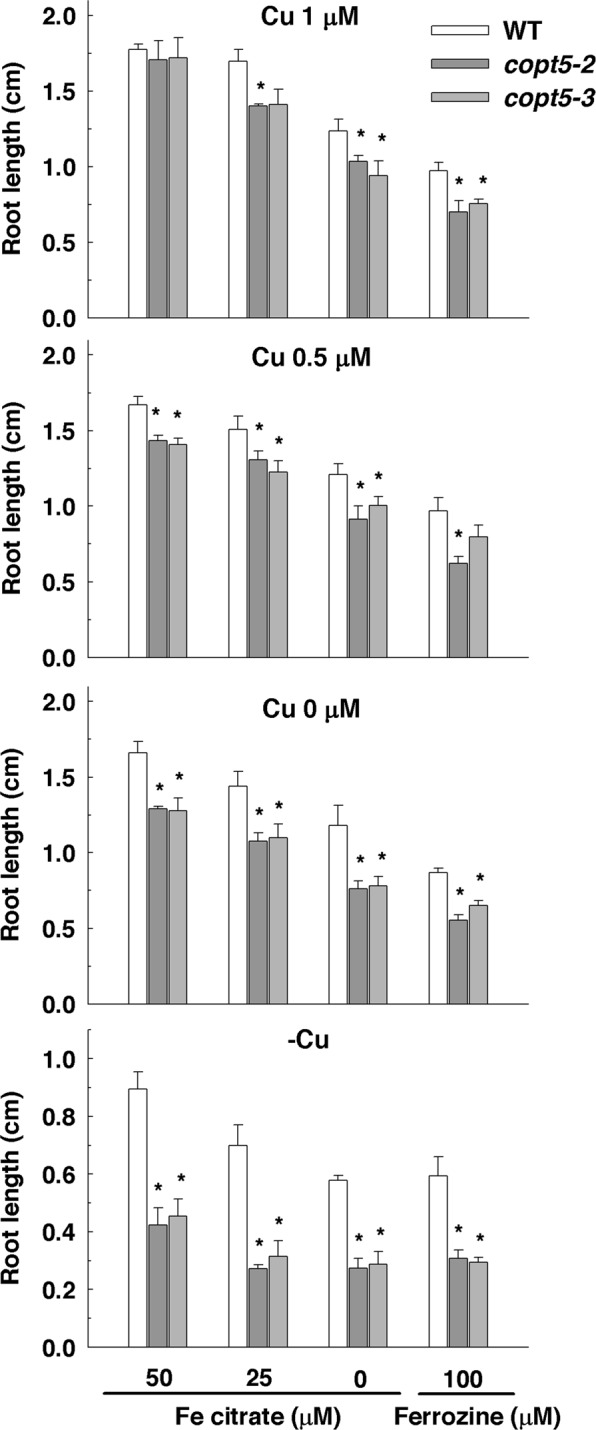
Root length of the *copt5* seedlings grown at different Cu and Fe availabilities. Root length of the 7 day-old plants WT, *copt5-2* and *copt5-3* seedlings grown under Fe deficiency (Ferrozine 100 µM), Fe mild deficiency (Fe-citrate 0 µM and Fe-citrate 25 µM) and Fe sufficiency (Fe-citrate 50 µM), and in the presence of Cu sufficiency (1 µM Cu), mild Cu deficiency (0.5 and 0 µM Cu) and severe Cu deficiency (100 µM BCS; -Cu). *Indicates statistical differences (P < 0.05) according to Tukey´s test. Bars are means ± SD of three replicates of at least 15 plants.

To further dissect a putative COPT5-mediated Cu-Fe crosstalk, we examined the development of WT and *copt5* seedlings at variable Cu and Fe conditions (Fig. 4). Under the control conditions, the root length of the WT and *copt5* plants was indistinguishable. As formerly reported^15^, the *copt5* mutant is more sensitive to severe Cu deficiency (-Cu) than WT seedlings by showing a 50% reduction of root length compared to the WT, and regardless of Fe concentration (Fig. 4, bottom panel). *copt5* is also more sensitive than the WT to Fe deficiency by displaying reduced root length according to Cu availability (Fig. 4).

To elucidate whether the involvement of COPT5 in the regulation of the Cu-Fe crosstalk was specific of a vacuolar Cu transporter, we compared the root lengths of the WT, *copt5* and plasma membrane *copt* mutants (*copt1*, *copt2*, *copt6* and *copt1copt2copt6*) grown under the control, Cu deficiency and Fe deficiency conditions (Fig. S4). Under Fe deficiency, only the *copt5* mutant was affected and it displayed a significantly exacerbated sensitivity to Fe shortage (Fig. S4).

### The vacuolar Fe transporter *nramp3nramp4* mutant is highly sensitive to Cu deficiency

**Figure 5:**
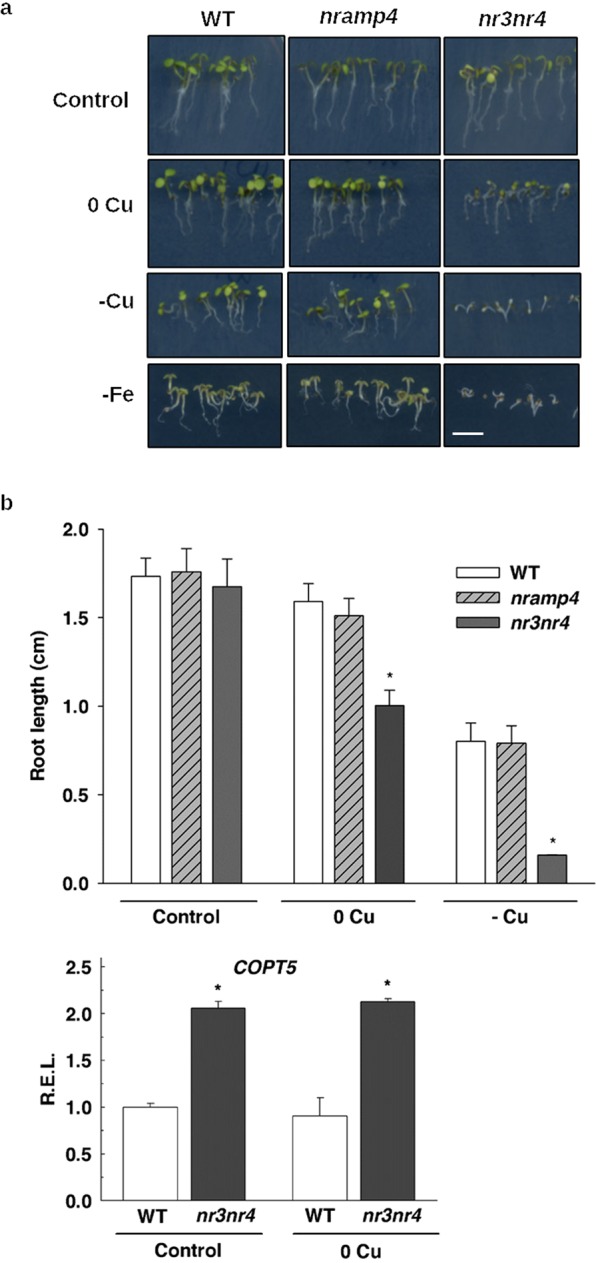
Characterisation of the *nramp4* and *nramp3nramp4* seedlings under Cu deficiency. (**a**) Photographs of the 7 day-old WT, *nramp4* and *nramp3nramp4* (*nr3nr4*) seedlings grown under Cu sufficiency (Control), mild (0 Cu), severe Cu deficiency (-Cu) and Fe deficiency (-Fe). Bar scale corresponds to 1 cm. (**b**) Root length of the plants shown in (**a**) except -Fe. *Indicates statistical differences (P < 0.05) according to Tukey’s test. Bars are means ± SD of three replicates of at least 15 plants. (**c**) Analysis of the *COPT5* relative expression levels (R.E.L.) in the 7 day-old WT and *nr3nr4* seedlings grown under Cu sufficiency (Control) and Cu scarcity (no added CuSO_4_; 0 Cu). Bars correspond to arithmetic means (2^−∆∆Ct^) ± SD (n = 3). *Indicates statistical differences (P < 0.05) in relation to the WT and *nr3nr4* mutant in each condition.

Similarly to COPT5, but in relation to Fe instead of to Cu, NRAMP4 is a vacuolar Fe transporter induced by Fe deficiency that is involved in Fe starvation responses^18^. By taking into account that *NRAMP4* expression is differentially regulated in the *copt5* mutant depending on Cu status (Fig. 2), we wondered whether the *nramp4* knockout mutant might be sensitive to Cu availability in the growth media. The *nramp4* mutant was grown under metal sufficiency (Control), and also under mild (0 Cu), severe Cu deficiency (-Cu) and Fe deficiency (-Fe), and displayed no evident growth defects (Fig. 5a,b). NRAMP3 has been described as being functionally redundant with NRAMP4 and, therefore, the *nramp3nramp4* double mutant showed a stronger phenotype under Fe deficiency^18^. Accordingly, *nramp3nramp4* was found to be highly sensitive to mild and severe Cu deficiency by showing a drastically reduced root length (31% and 83% reduction compared to the WT, respectively) (Fig. 5a,b).

Although *NRAMP4* was induced under Cu deficiency conditions in the *copt5* seedlings (Fig. 2), *NRAMP3* showed no statistical differences between genotypes, although its expression was enhanced by Cu deficiency in both the WT and *copt5* seedlings (Fig. S5a). In addition, *COPT5* expression increased in the *nramp3nramp4* mutant regardless of the Cu availability in the medium (Fig. 5c). We also assessed the effect of Cu deficiency on the *pNRAMP4::GUS* spatial expression pattern (Fig. S5b). The *NRAMP4* promoter drove *GUS* expression to roots under both Cu and Fe deficiency. In accordance with *COPT5*, *NRAMP4* expression was also concentrated on the crown of seedlings under Fe deficiency.

### Superoxide dismutase metalloprotein substitution does not account for the metal mobilization in the *copt5* mutant

**Figure 6:**
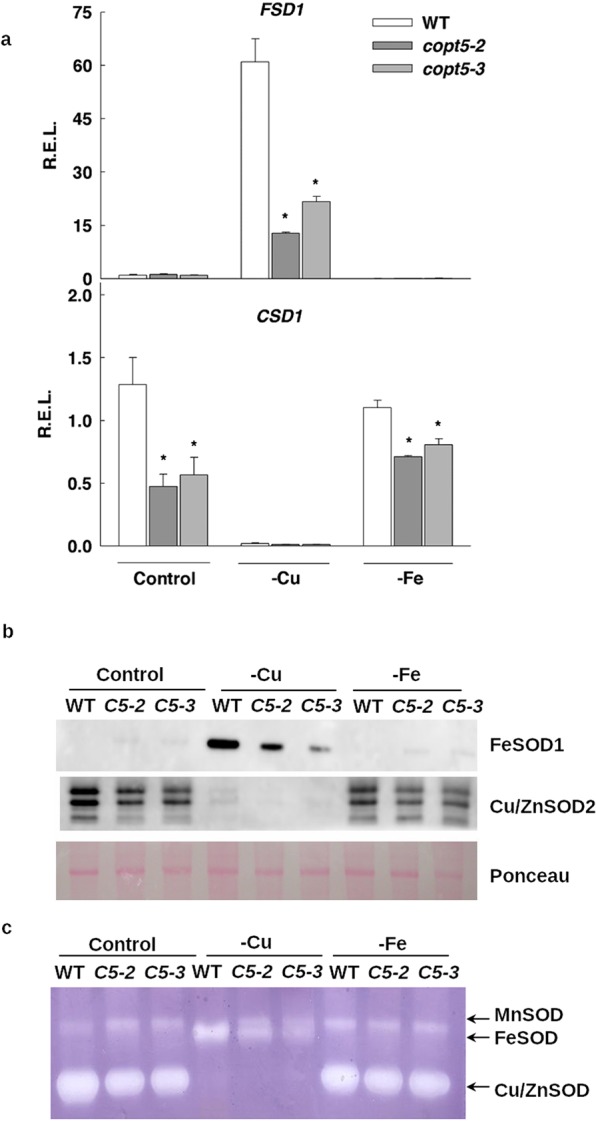
Effect of Cu availability on SOD regulation in the *copt5* mutants. (**a**) The *FSD1* and *CSD1* relative expression levels (R.E.L.) in relation to the WT under control conditions. The qRT-PCR analysis done on the 7 day-old WT, *copt5-2* and *copt5-3* seedlings grown under the same conditions used in Fig. 3. Bars correspond to arithmetic means (2^−∆∆Ct^) ± SD (n = 3). *Indicates statistical differences (P < 0.05) in relation to the WT and *copt5* mutants in each condition. (**b**) SOD immuno-detection. Soluble protein extraction was performed with the 7 day-old WT, *copt5-2* (C5–2) and *copt5-3* (C5–3) seedlings grown under the same conditions used in Fig. 3. Immuno-detection of FSD1 and CSD2 using 35 µg of protein extract. Ponceau staining is shown as a loading control. For the complete blots, see Fig. S10. (**c**) The SOD enzyme activities analysed in the native gels loaded with 100 µg of protein extract. The gel was stained for total SOD activity. Full-length blots/gels and replicates are presented in Supplementary Figure S9.

The best-known example for the Cu-Fe interaction is SODs metalloprotein substitution. While *FSD1*, which encodes the FeSOD, is expressed under Cu deficiency, *CSD2* and *CSD1*, which respectively encode chloroplastic and cytosolic Cu/Zn SODs, are mostly expressed under Cu sufficiency^6^. We analysed the SODs expression in the WT and *copt5* mutant at different Cu and Fe concentrations in the media (Figs 6a, S6a). In agreement with the exacerbated Fe deficiency experienced in the *copt5* mutant, *FSD1*, *FSD2* and *FSD3* expressions were lower in the mutant than in the WT subjected to Cu deficiency (Figs 6a, S6a). Regarding *CSDs*, a decrease in *CSD1* and *CSD2* mRNAs were measured in the *copt5* mutants in relation to the WT under metal sufficiency (Figs 6a, S6a). Moreover, *CSD1* expression lowered in the *copt5* mutant under Fe deficiency (Figs 6a). Altogether, these results indicate a defect in SOD substitution as both SODs levels were lower in the *copt5* mutant.

Next we assessed the accumulation of SOD proteins by immunoblot (Fig. 6b) and tested their enzymatic activity with gel assays (Fig. 6c). The FeSOD1 protein content and FeSOD activity in gels decreased in the *copt5* mutant under Cu deficiency conditions, which agrees with the mRNA accumulation pattern (Fig. 6a). Regarding Cu/ZnSOD, and according to the mRNA accumulation, a low level of protein and activity were also observed in the *copt5* mutants compared to the WT under Cu sufficiency and Fe deficiency (Fig. 6). Therefore, these data suggest that the *copt5* mutant has impaired SOD substitution which might result in exacerbated oxidative stress in this genotype.

Next SOD expression was examined in the *nramp3nramp4* mutant (Fig. S7). Similarly to the results observed in the *copt5* mutant, Cu/ZnSODs were reduced in the *nramp3nramp4* mutant under Cu suficiency. In contrast, the *FSDs* transcript levels did not decrease under Cu deficiency conditions in this mutant (Fig. S7a). However, the protein level and SOD activity showed an unexpected result under the control conditions. Cu/ZnSOD protein content and activity were not detected in the *nramp3nramp4* mutant. FeSOD activity and protein accumulation (Fig. S7b,c) did not correlate with the transcript levels (Fig. S7a). These results deserve further reasearch in order to understand the putative post-transcriptional responsive mechanisms operating in FeSOD activity in the *nramp3nramp4* mutant.

To summarise, under the experimental conditions used herein, the regulation of SOD activity was defective in both the *copt5* and *nramp3nramp4* mutants (Figs. 6, S6, S7). Whereas FeSOD activity in the *copt5* mutant was lower than the WT under Cu deficiency conditions, its activity in the *nramp3nramp4* mutant was present under the control conditions. Despite the described defects, these results exclude the possibility of attributing the differential use of metals in the *copt5* and *nramp3nramp4* mutants for the corresponding SOD substitutions.

### The COPT5 function affects Fe localisation and consumption during the germination process

**Figure 7:**
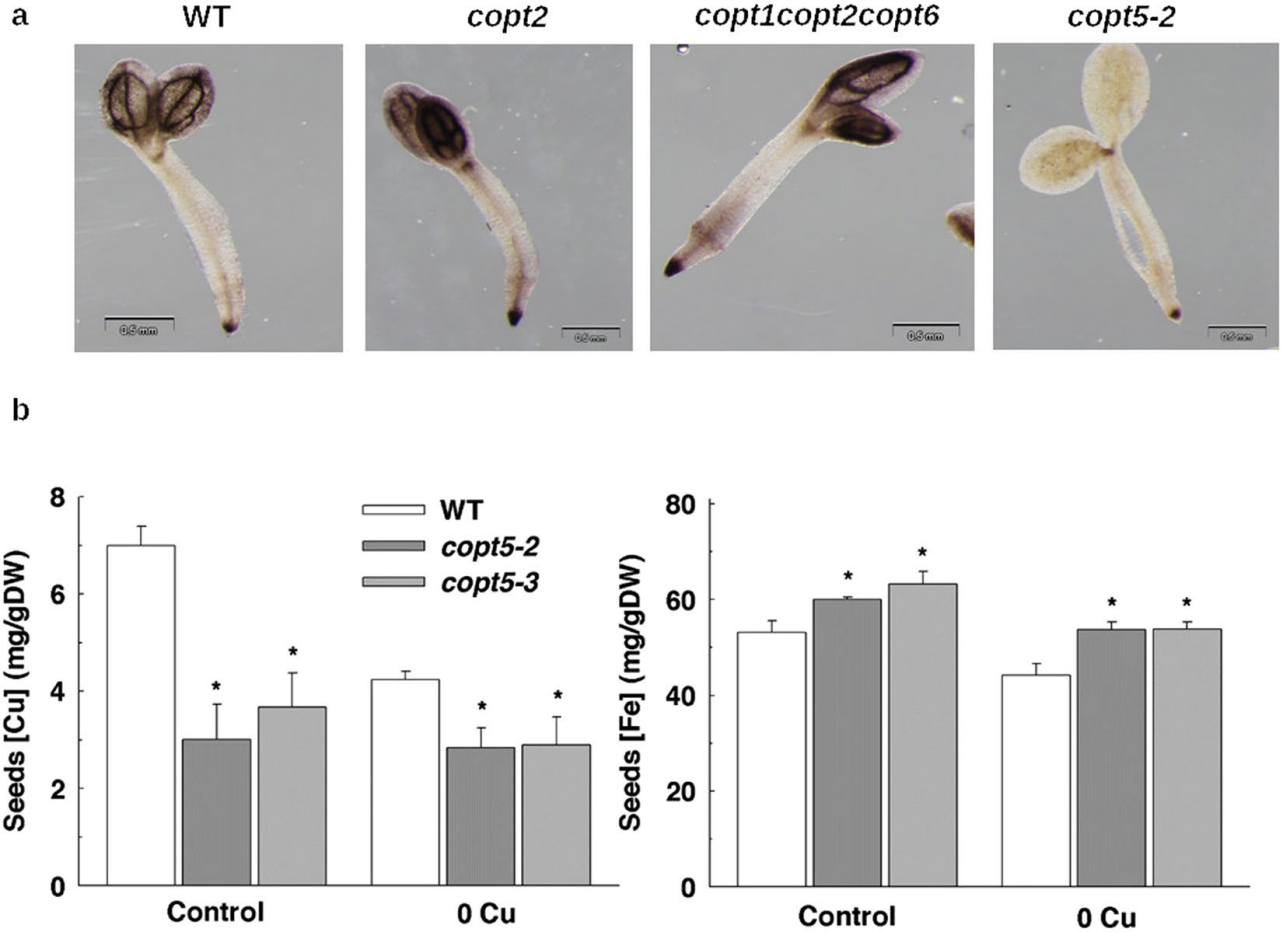
Fe localisation in the *copt* seedlings and metal concentrations in *copt5* seeds. (**a**) Perls/DAB staining of the 2 day-old WT, *copt2*, *copt1copt2copt6* and *copt5* seedlings grown in ½ MS liquid medium. Fe staining appears in black. (b) Cu and Fe contents in the seeds harvested from adult plants watered with Hoagland’s solution with 0.01 µM CuSO_4_ (Control) or without Cu supplementation (0Cu). *Indicates statistical differences (P < 0.05) according to the t-test. Bars are means ± SD of three replicates of 15 mg of dry weight from seed.

Metal reallocation could be another factor that explains the altered Cu-Fe interaction observed in the *copt5* mutant. Since *copt5* plants exhibit defects in Cu long-distance transport^16^, we wondered if they also had problems in Fe translocation. In order to follow the fate of Fe in the *copt5* mutant, we used Perls/DAB staining on 3-day-old seedlings, as Fe is rapidly remobilised in plants after the germination process^39^. A lower Fe in seedlings was observed in the *copt5* mutant compared to the WT, *copt2* and *copt1copt2copt6* mutants in the 3-day-old seedlings (Fig. 7a), which suggests a connection between the vacuolar Cu transport and Fe distribution.

Metal contents were determined in the WT and *copt5* seeds harvested from the plants grown in the greenhouse with different Cu supplies (Fig. 7b). In general, both Cu and Fe contents were lowered under Cu deficient conditions. As expected, the *copt5* seeds contained less Cu than the WT seeds, and regardless of Cu availability in the medium. However, Fe accumulation was slightly higher in the *copt5* seeds compared to the WT (Fig. 7b).

**Figure 8:**
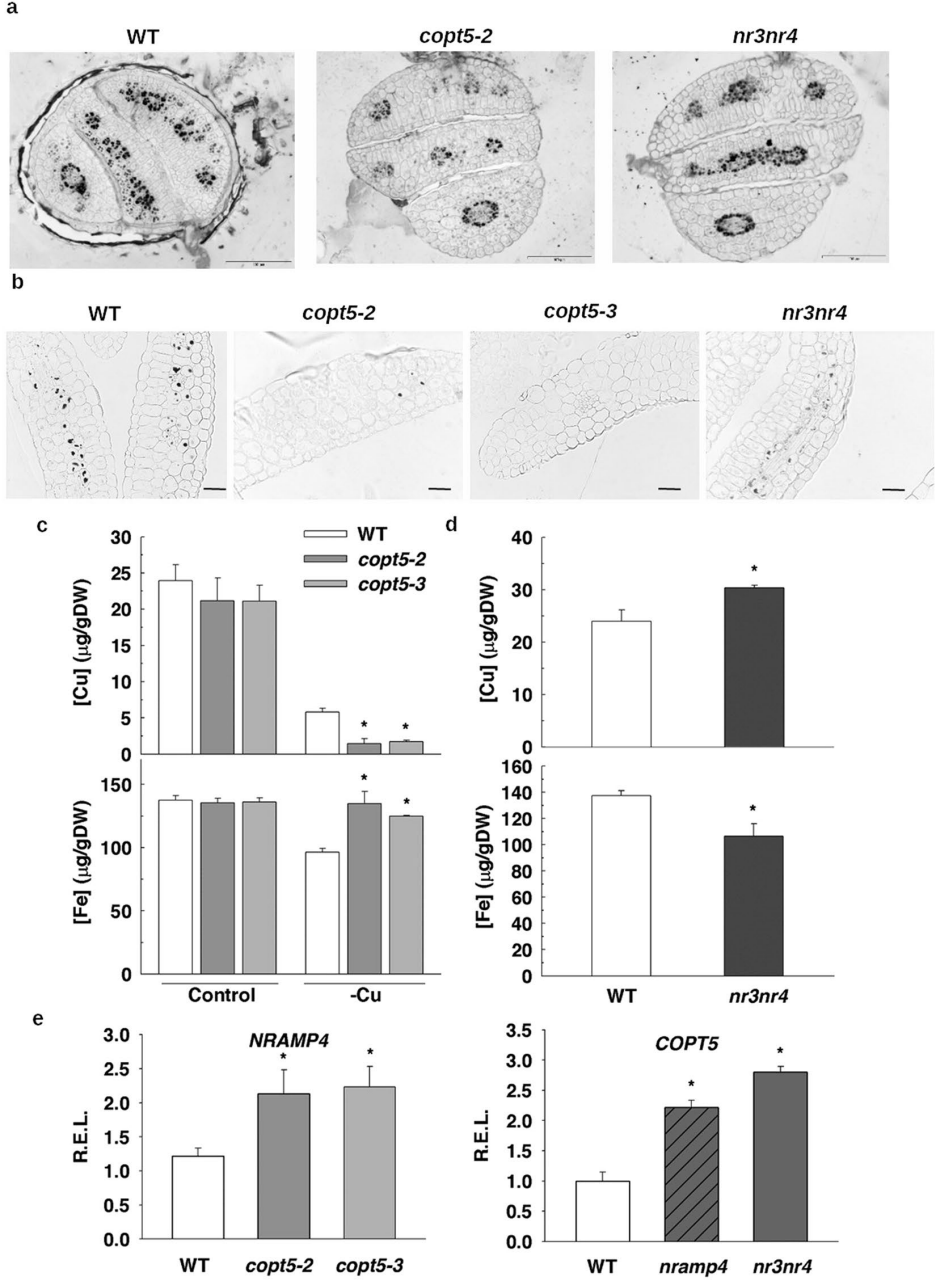
Fe localization and metal content in the *copt5* and *nramp3nramp4* mutants. (**a**) Perls/DAB staining of the dry seeds from WT, *copt5-2* and *nramp3nramp4* (*nr3nr4*). (**b**) Perls/DAB staining of the cotyledon sections from the 3 day-old seedlings from WT, *nramp3nramp4* (*nr3nr4*)*, copt5-2* and *copt5-3* grown under Cu sufficiency control conditions. Cu and Fe contents in the cotyledons from *copt5* (**c**) and *nramp3nramp4* (*nr3nr4*) (**d**) from the seeds grown as mentioned in the Methods section. (**e**) The relative expression levels (R.E.L.) of the *NRAMP4* and *COPT5* genes were determined by qRT-PCR in the 3 day-old WT seedlings grown under the control conditions in the *copt5-2* and *copt5-3* mutants (left) and the *nramp4* and *nramp3nramp4* (*nr3nr4*) mutants (right). The *mRNA* levels are expressed as relative expression in relation to the WT under control conditions. Bars correspond to arithmetic means (2^−∆∆Ct^) ± standard deviation (SD) (n = 3). *Indicates statistical differences (P < 0.05) between the values of the WT and the mutants in each condition.

Therefore, the *copt5* mutant showed enhanced Fe content in seeds (Fig. 7b), but lacked Fe staining in the 3-day-old seedlings (Fig. 7a). To better understand this fact, we first checked the localisation of Fe in dry seed embryos (Fig. 8a), where a similar Fe pattern was displayed in the WT and in the *copt5* and *nramp3nramp4* mutants. Secondly, Fe staining was detected in cotyledons after 3 days of post-germinative growth (Fig. 8b). Fe is visualised in the WT as spots, which are the vacuoles where stored, and in the *nramp3nramp4* mutant, where Fe remained localised in the vacuoles because it was unable to remobilise the metal^39^. However, less Fe staining was detected in the vacuoles of the *copt5* mutant (Fig. 8b) despite it being present in the embryo vascular bundles (Fig. S8a). *COPT5* expression was also assessed in embryos by a GUS assay (Fig. S8b). These results suggest that the consumption of vacuolar Fe was accelerated in the *copt5* mutant and that the COPT5 function might play a role in Cu-dependent Fe mobilisation during the germination process.

Next we analysed both Cu and Fe content in the cotyledons from the seedlings grown under different Cu and Fe conditions (Fig. 8c). Whereas the *copt5* cotyledons showed lower Cu content under Cu deficiency, they contained more Fe (Fig. 8c) despite the *copt5* mutant exhibiting exacerbated sensitivity to Fe deficiency (Fig. 4). The metal content analysis in the *nramp3nramp4* mutant showed the opposite pattern (Fig. 8d). Whereas Cu content was higher in the mutant than in the WT cotyledons, Fe content lowered in the *nramp3nramp4* mutant (Fig. 8d), which suggests the Fe-Cu interconnection functioning in both ways. This interdependence of both metals was further illustrated at the gene expression level as *NRAMP4* expression was significantly induced in the 3-day-old *copt5* mutant seedlings, as well as *COPT5* is in the double *nramp3nramp4* mutant (Fig. 8e). These results matched the higher Fe content found in the *copt5* mutant seeds (Fig. 7b), and suggest that increased NRAMP-dependent Fe remobilisation from the vacuolar pools could perhaps serve for extracellular translocation.

## Discussion

Extensive studies on Fe deficiency responses in plants have been reported^28,40,41^ but understanding how plants acclimate to low Fe levels under other metal deficiencies, such as Cu, remains obscure. The mechanistic insight into how Fe and Cu homeostases are intertwined at the subcellular and whole plant levels, the variety of the affected processes and the physiological significance of their interactions have only begun to be explored^3–5,14^. One strategy to address the crosstalk of metals is to use the plant mutants impaired in the specific metal transport, such as the high affinity Cu transport proteins of the COPT family, and to look for effects on other metals’ homeostasis^14,17^.

In the present study, we show that whereas the mutants of the high affinity Cu transporters located at the plasma membrane (*copt1*, *copt2*, *copt6*) are not sensitive to Fe deficiency, the impairment in the function of the tonoplast COPT5 protein^15,16^ clearly affects Fe localisation and several Fe deficiency responses (Figs. 7a, S2). Hence the different localisation of COPT2 and COPT5 at external and internal cellular membranes, respectively^13–16^, could explain the diverse Fe-deficiency phenotypes observed in the *copt2* and *copt5* mutants. While a *copt2* mutant is more resistant than WT plants to the chlorosis induced by simultaneous Fe and Cu deficiencies^14^, the *copt5* mutant is more sensitive to Cu and Fe availabilities (Fig. 4). This fact illustrates the differential role of internal Cu pools, versus the external Cu content (at the apoplast and the media) regarding the Cu crosstalk with Fe homeostasis. Accordingly, *COPT2* and *COPT5* are up- and down-regulated under Fe deficiency, respectively^14^, which underscores the complex regulation of Cu homeostasis under low Fe.

Biological processes, including responses to hormones, lipid metabolism, responses to toxin and transition metal ion transport, are affected in *copt5* mutants under a low Cu supply (Table S2). Of the transition metal ion transporters, *NRAMP4* was highly induced in the *copt5* mutant under Cu deficiency (Table 1, Fig. 2). As both NRAMP4 and COPT5 were located in the tonoplast, and *NRAMP4* expression was up-regulated in the *copt5* mutant under Cu deficiency, we further studied the *NRAMP4* response to Cu scarcity. Although the presence of five GTAC elements in the *NRAMP4* promoter (not shown) was compatible with increased expression under Cu deficiency mediated by the SPL7 transcription factor, *NRAMP4* expression under Cu deficiency is independent of SPL7 according to published global analysis data^5^. Arabidopsis NRAMP3 and NRAMP4 are able to transport Fe, Mn and Cd, and also Zn in the case of NRAMP4^18,42,43^. However, Cu levels were not affected, at least not in the *nramp3* mutant^42^. Based on these results, the modification of Cu transport as a result of increased *NRAMP4* expression in seedlings seems unlikely. Instead a subsequent NRAMP4-dependent increase in the remobilisation of other metals, such as Fe, from vacuoles probably takes place in the *copt5* mutant. In return, *COPT5* expression was induced in the *nramp3nramp4* mutant (Fig. 8), which suggests enhanced COPT5-dependent Cu remobilisation from the vacuoles in the *nramp3nramp4* double mutant as COPT transporters are specific for Cu^+^ ^44,45^. The *nramp3nramp4* mutant is even more sensitive to Cu deficiency than the *copt5* mutant. Whereas the root length of the *copt5* mutant reduced by approximately 50% under severe Cu deficiency (Fig. 4), the decrease in the *nramp3nramp4* mutant was more than 80% under only Cu scarcity (Fig. 5). Moreover, the *GUS* expression driven by the *COPT5* and *NRAMP4* promoters indicated that both genes were expressed in the vascular bundles under Cu deficiency (Figs. 3 and S5b). This Fe and Cu interconnection was not based on the well-established SOD metalloprotein substitution^4,6^ as the *mRNA* levels, and the Cu/ZnSOD, FeSOD protein and activity levels, were always lower in the *copt5* mutant, independently of the metal status (Fig. 6). Taken together, these results suggest that Cu and Fe vacuolar pools are interconnected, and in such a way that the lack-of-function in a tonoplast metal transport protein drives the remobilisation of the other metal by inducing the expression of the corresponding vacuolar transporter. Perhaps the aim is to long distance metal transport and translocation from roots to the aerial parts.

*OPT3* and *YSL1* regulation in the *copt5* mutant (Fig. 2) agreed with the recently suggested roles of OPT3 and YSL1 in the redistribution of Fe in phloem and the surrounding parenchyma cells^37^. Our data suggest that *COPT5* repression under Fe deficiency (Fig. 3) possibly aims to avoid xylem Cu loading as the higher Cu affinity for common metal chelators^9^ could further limit Fe delivery to upper organs. Hence Fe deficiency might involve a compromise for cells as Cu could help to alleviate certain Fe functions, but Cu transport in the xylem could further limit Fe delivery to aerial parts. In agreement with this idea, an increased Fe content in sink organs, such as seeds, was observed in the *copt5* mutant (Fig. 7b). Moreover, Fe levels significantly rose in the *copt5* cotyledons under Cu deficiency compared to the WT (Fig. 8c). Accordingly, altered Cu distribution and Cd translocation have been observed in the *copt5* mutant^16,17^, which further confirms the influence of the COPT5 function on the long-distance transport of metals. These results encourage further research that aims to increase Fe contents in edible parts of horticultural crops, and to set the basis for future biotechnological improvements to produce Fe/Cu biofortified food^46^.

This report provides compelling evidence for the interaction between vacuolar Fe and Cu pools under metal deficiencies. This fact become particularly more evident in the *copt5* and *nramp3nramp4* mutant backgrounds, which are unable to retrieve Cu and Fe, respectively, from vacuoles^15,16,18^. Collectively, these results underline that subcellular trafficking and ROS signalling might contribute to the complexity of the interaction between Cu and Fe deficiency responses.

## Methods

### Plant growth conditions

*Arabidopsis thaliana* ecotype *Columbia* (Col-0) was used as the control wild type (WT). The *pCOPT5::GUS* and *pNRAMP4::GUS* plants, the *copt5-2, copt5-3* and *nramp3nramp4* knock-out mutants, the complemented *COPT5* (*pCOPT5::COPT5::GFP*) and the overexpressor *COPT5*^*OE*^ (*pCaMV35S::COPT5::HA*) lines have all been previously described^15,18^. To determine the metal content in seeds, plants were sown in soil pots and grown under greenhouse conditions. Plants were watered with tap water for 20 days, after which time Cu treatments commenced using Hoagland’s solution with 0.01 µM CuSO_4_ (Control) or without Cu supplementation (0 Cu). For growth on plates, seeds were surface-sterilised with sequential washes in 70% ethanol (5 min), bleach (5 min) and water (2 × 2 min) before being resuspended in agar 0.1% (w/v) and sown on plates containing ½ MS (Murashige and Skoog) medium supplemented with 1% sucrose (w/v). Unless otherwise indicated, ½ MS with 1 µM CuSO_4_ and 50 µM Fe citrate were used for the metal sufficiency control conditions (Control), and ½ MS with 100 µM BCS and/or 100 µM ferrozine, Cu and Fe chelators, respectively, provided the severe deficiency growing conditions (-Cu and -Fe). Cu deficiency was obtained by home-made ½ MS with no added CuSO_4_ (0Cu). In all cases, intermediate photoperiodic conditions (12 h light, 20–23 °C/12 h darkness, 16 °C) were applied. Root length was measured by the *Image J 1.42 q software* (http://rsb.info.nih.gov./ij).

### Metal content determination

The fresh *Arabidopsis* material was washed once with 2 mM CaSO_4_ and 20 mM EDTA and 3 times with MilliQ H_2_O, before being dried at 65 °C for 2 d and digested with 65% (v/v) HNO_3_ and H_2_O_2_ 30% (v/v) at 140 °C. The digested samples were then diluted with Millipore H_2_O (*Purelab Ultra*), and the Cu and Fe contents were determined by microwave-plasma atomic emission spectroscopy (MP-AES Agilent technologies) at the *Institute National de la Recherche Agronomique* (INRA) (Montpellier, France) using the manufacturer’s standard solutions for the calibration curves.

### Gene expression analysis by real-time quantitative PCR

The total RNA extraction, reverse transcription and qRT-PCR analyses were performed as described^47^. The forward (F) and reverse (R) sequences for specific primers are shown in Table S1. To transform fluorescent intensity measurements into relative mRNA levels, a 2-fold dilution series of a mixture that contained an equal amount of each cDNA sample was used and standard curves were constructed for all the studied genes. The *UBIQUITIN10* reference gene was used for data normalisation. Each sample was analysed in triplicate, and the mean ratios ± SE were calculated.

### Immunodetection and SOD activity

Soluble 7-day-old seedlings proteins were extracted for the SDS polyacrylamide and non-denaturing gel analysis^48^. Total protein was quantified according to the Bradford method^49^ using bovine serum albumin as a standard. For the immunodetection analysis, 35 µg of protein extract were loaded into 15% SDS polyacrylamide gels. The antibodies used for Cu/ZnSOD2 and FeSOD1 were obtained from Agrisera (Agrisera AB, Vännäs, Sweeden). For SOD isoenzyme separation and activity purposes, 100 µg of protein extract were loaded into 15% non-denaturing polyacrylamide gels, which were then stained for activity as previously described^48,50^. Each experiment was repeated 5 times with identical results. Representative gels are shown.

### Perls/DAB staining and histological procedures

For organ staining, the seedlings were vacuum-infiltrated with equal volumes of 4% (v/v) HCl and 4% (w/v) K-ferrocyanide (Perls stain solution) for 15 min and incubated for 30 min at room temperature^34^, followed by the DAB intensification^39^. The roots of the 3-day-old seedlings were previously rinsed with EDTA and distilled water. The fixed samples were washed with 0.1 M Na-phosphate buffer (pH 7.4) 3 times, and dehydrated in successive baths of 50, 70, 90, 95 and 100% ethanol, butanol/ethanol 1:1 (v/v) and 100% butanol. Then the tissues were embedded in the Technovit 7100 resin (Kulzer) according to the manufacturer’s instructions and thin sections (3 µm) were sliced. Sections were deposited on glass slides and incubated for 45 min in Perls stain solution, when the intensification procedure was applied^39^. The pictures of the cross sections were obtained by an Olympus BX61 microscope and the Cell-A software was used. For the pictures of whole seedlings, an Olympus SZX16 stereoscopic microscope was used.

### GUS assay

The seedlings and organs of the adult *pCOPT5::GUS* and *pNRAMP4::GUS* plants were embedded with the substrate solution [100 mM NaPO_4_, pH 7.2, 0.5 mM K_3_Fe(CN)_6_, 0.5 mM K_4_Fe(CN)_6_, 0.1% (v/v) Triton X-100, 0.5 mM 5-bromo-4-chloro-3-indolyl-β-D glucuronide (AppliChem), and 10 mM EDTA, pH 7.2]^51^. Reactions took place at 37 °C and were stopped with ethanol (70%). The pictures of the cross sections were obtained using the Olympus AT70F microscope and the Infinity software.

### Microarray analysis

Three biological replicates of the 7-day-old seedlings of the WT and *copt5-2* plants grown during a 12 h light/12 h dark photoperiod were used for each treatment. The Cu-deficient medium was supplemented with 100 *μ*M BCS (-Cu), whereas Cu sufficiency was obtained by adding 1 µM CuSO_4_ (Control). Total RNA was isolated with the RNeasy Plant Mini Kit (Qiagen), and antisense RNA was amplified using the MessageAmp II aRNA Amplification kit (Ambion). The *Arabidopsis* (V4) Gene Expression Microarray 4 × 44 K (Agilent Technologies) was hybridised by the technical services of the *Instituto de Biología Molecular y Celular de Plantas* (IBMCP, UPV-CSIC, Valencia, Spain). The expression values (log_2_), data normalisation and statistical analyses were obtained by the Genespring GX microarray analysis software (Agilent Technologies). The differential expressed genes (DEG) were identified by applying a false discovery rate (FDR) below than 1% and 1.5-fold change (log_2_ |1.5|). FatiGO+ (Babelomics, http://bioinfo.cipf.es/)^52^ was used to identify biological processes that were significantly under- or over-represented.

### Statistical analyses

The statistical differences in the gene expression analyses were identified by the pair-wise fixed reallocation randomisation test (P < 0.05)^53^. For the other parameters, one-way ANOVAs were performed. Significant differences between means were established after *post hoc* tests (Tukey or Games-Howell, according to data homoscedasticity; P ≤ 0.05) using version 19.0.0 of the IBM SPSS Statistics software. Data are provided as the mean values ± SD of the different biological samples used in each experiment, as indicated in the figure legends.

## Supplementary information

Supplemental Material

## Data Availability

The microarray raw data were deposited in the National Center for Biotechnology Information Gene Expression Omnibus^54^ and are accessible through accession number GSE91044. The microarray data were validated by RT-qPCR gene expression analyses on selected genes^55^. The materials used herein this work will be available from the authors upon reasonable request, and in accordance with the Journal policy described in the Instructions for Authors (Availability of materials and data).
