# Supplementary material for: Copper transporter COPT5 participates in the crosstalk between vacuolar copper and iron pools mobilisation
Source: Sci Rep. 2019 Mar 15;9:4648. doi: 10.1038/s41598-018-38005-4 (PMC6420658; doi:10.1038/s41598-018-38005-4)
Supplement: Supplementary file 1 — Supplemental Material [file 41598_2018_38005_MOESM1_ESM.pdf]

Supplementary Information

Copper transporter COPT5 participates in the crosstalk between vacuolar copper and iron pools mobilisation

Àngela Carrió-Seguí, Paco Romero, Catherine Curie, Stéphane Mari and Lola Peñarrubia

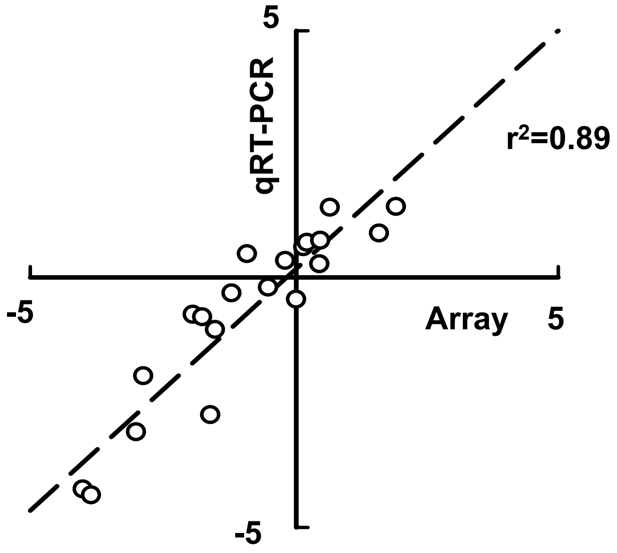

Fig. S1 Microarray data validation. Multiple linear regression analysis (r^2) of the selected genes was performed by comparing the qRT-PCR values and the *Arabidopsis* (V4) Gene Expression Microarray 4x44K (Agilent Technologies) data for all the studied genotypes and growing conditions. The graph shows the correlated values for all the genes in the same plot (r^2=0.89). Individual regression analyses for each gene revealed r^2 values that ranged from 0.69 to 0.99 (data not shown).

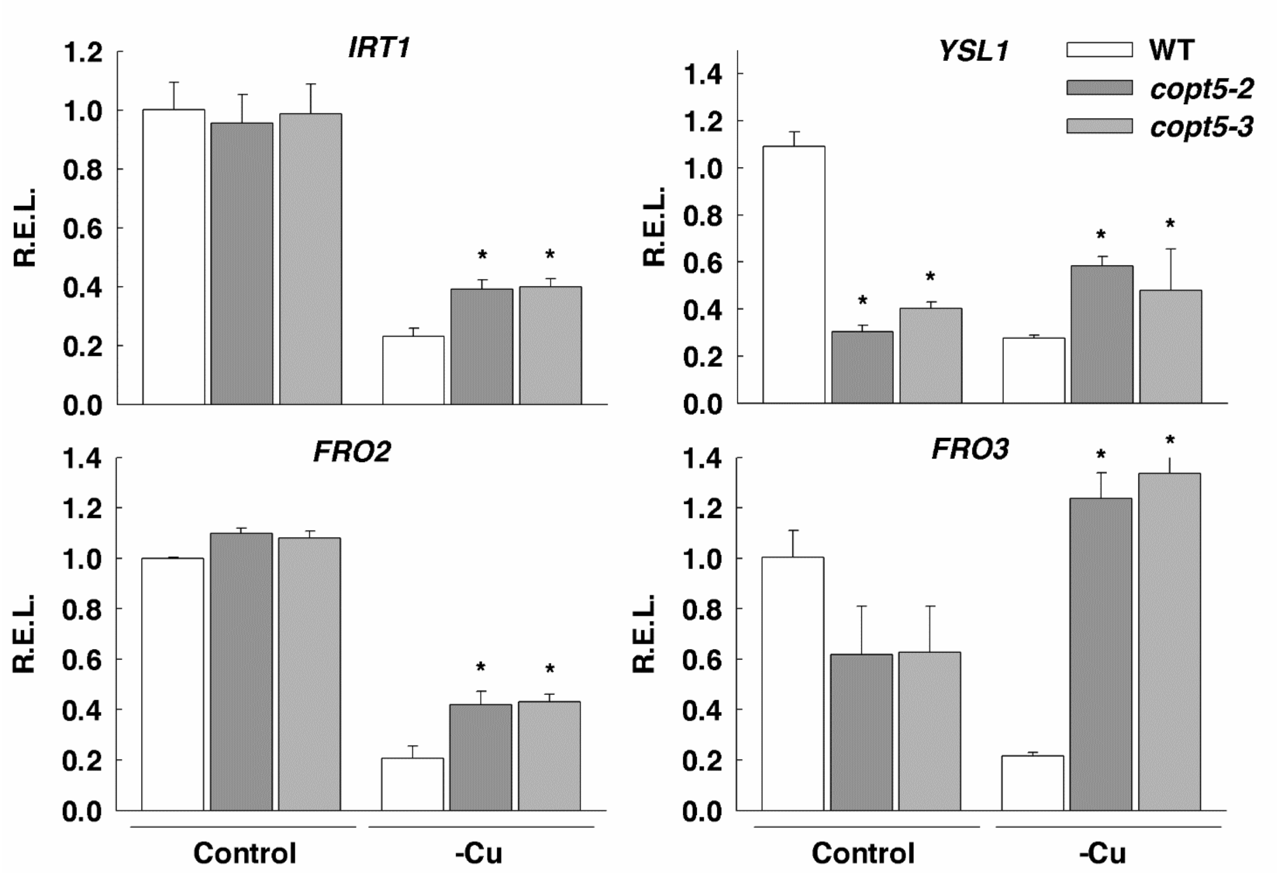

Fig. S2 Fe homeostasis-related expression in the WT and *copt5* mutants grown under metal deficiency. The relative expression levels (R.E.L) of the *IRT1, YSL1, FRO2 and FRO3,* genes were determined by qRT-PCR in 7-day-old WT seedlings grown under Cu sufficiency (Control) and Cu deficiency (-Cu) in WT (white bars) and *copt5 mutants* (grey bars). The *mRNA* levels are expressed as relative expression levels in relation to the WT under control conditions. Bars correspond to arithmetic means ($2^{-\Delta\Delta Ct}$) ± standard deviation (SD) (n=3). For each particular gene, * indicates statistical differences ($P < 0.05$) between the values of the WT and *copt5* mutants in each condition.

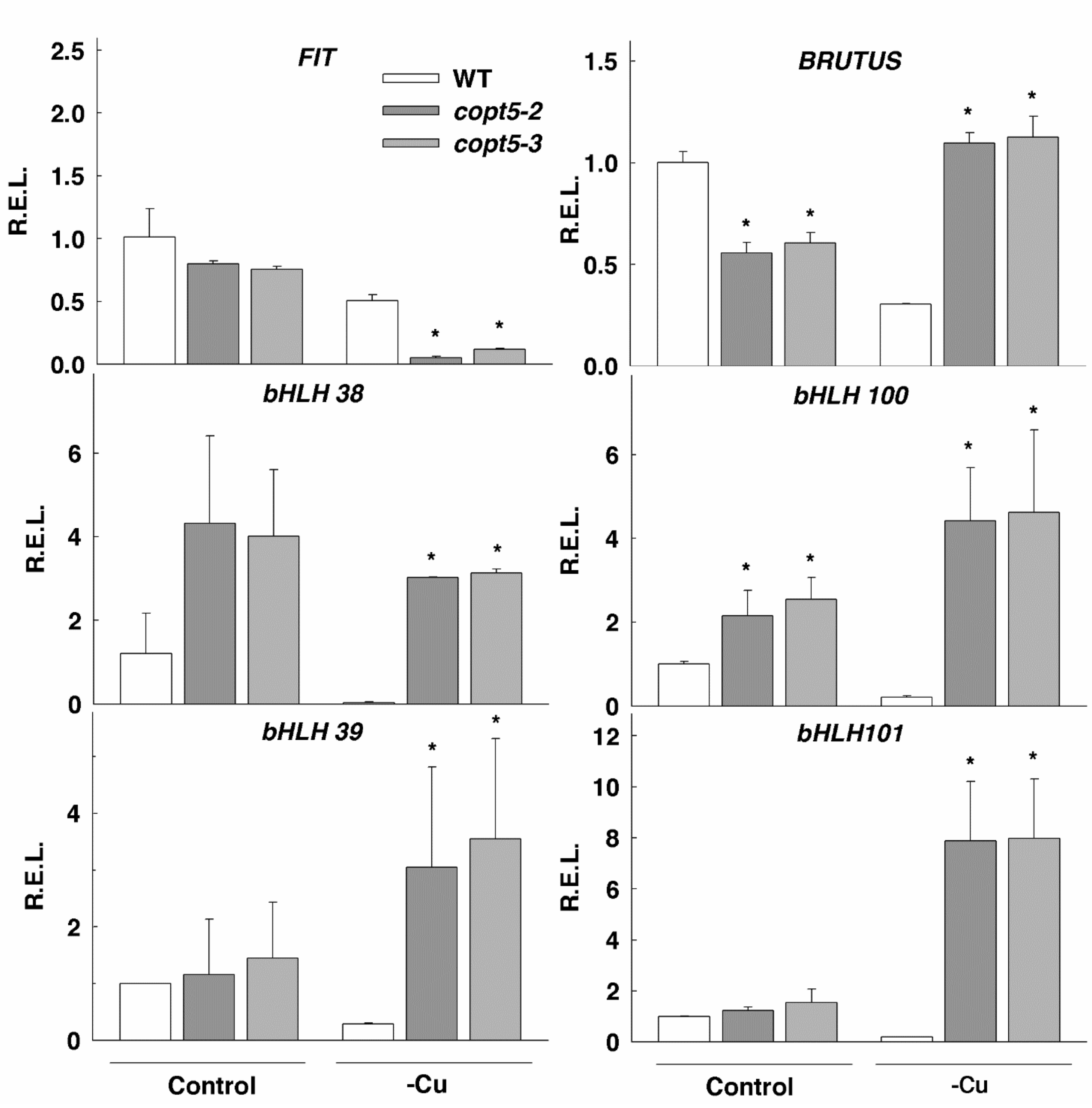

Fig. S3 Fe-related regulators of gene expression in the WT and *copt5* mutants. The 7-day-old WT (white bars) and *copt5* mutants (grey bars) seedlings grown under Cu sufficiency (Control) and Cu deficiency (-Cu). The relative expression levels (R.E.L.) of the *FIT*, *BRUTUS*, *bHLH38*, *bHLH100*, *bHLH39* and *bHLH101* genes was determined by qRT-PCR. The *mRNA* levels are expressed as as relative expression levels in relation to the WT under control conditions. Bars correspond to arithmetic means ($2^{-\Delta\Delta Ct}$) ± standard deviation (SD) (n=3). For each particular gene, * indicates statistical differences (P < 0.05) between the values of the WT and *copt5* mutants in each condition.

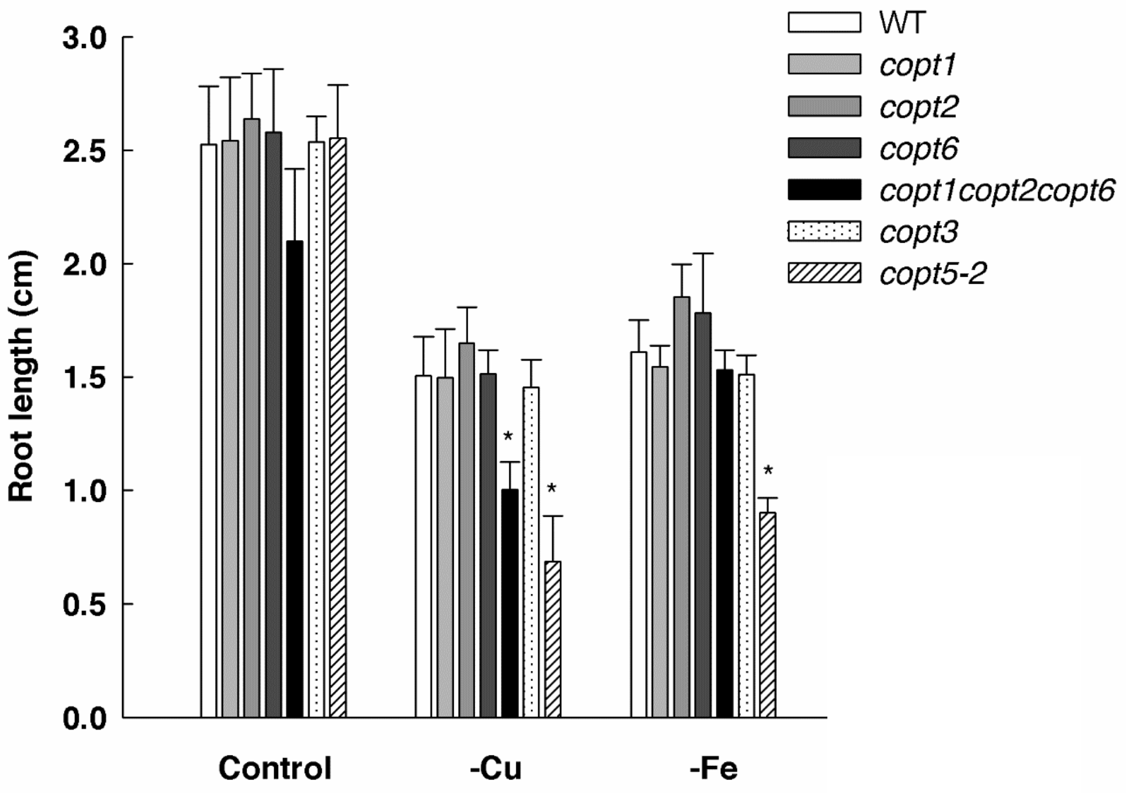

Fig. S4 Root length of the *copt* mutants grown at different Cu and Fe availabilities. Root length of the 7-day-old WT, *copt1*, *copt2*, *copt6*, *copt1copt2copt6* and *copt5* grown in ½ MS medium supplemented with 1 µM $CuSO_4$ and 50 µM Fe-citrate (Control), 100 µM BCS and 50 µM Fe-citrate (-Cu) and 1 µM $CuSO_4$ and 100 µM Ferrozine (-Fe). * indicates statistical differences ($P<0.05$) according to Tukey's test compared to the WT under the same condition.

a

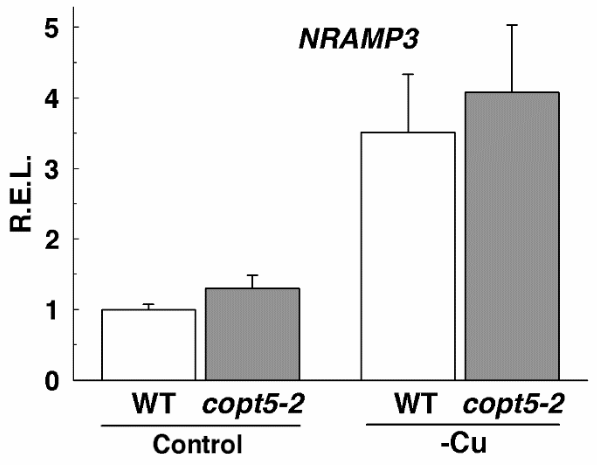

b

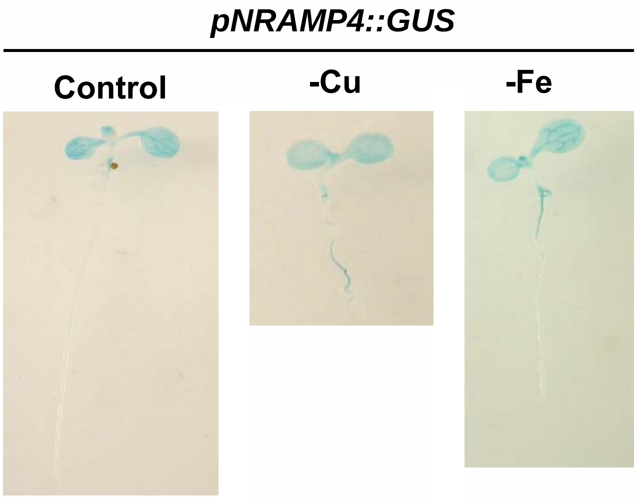

Fig. S5 *NRAMP3* and *NRAMP4* expressions at different metal availabilities. (a) *NRAMP3* expression in the 7-day-old WT and *copt5* grown in ½ MS medium supplemented with 1 µM $CuSO_4$ and 50 µM Fe-citrate (Control) and 100 µM BCS and 50 µM Fe-citrate (-Cu). The *mRNA* levels are expressed as relative expression levels in relation to the WT under control conditions. Bars correspond to arithmetic means ($2^{-\Delta\Delta Ct}$) ± standard deviation (SD) (n=3). For each particular gene, asterisks indicate statistical differences (P < 0.05) between the values of the the WT and *copt5* mutants in each condition. (b) The *NRAMP4* driven GUS expression. The 7 day-old *pNRAMP4::GUS* seedlings were grown under the same conditions indicated in (a) plus 1 µM $CuSO_4$ and 100 µM Ferrozine (-Fe). Representative pictures of the seedlings grown under each conditions are shown.

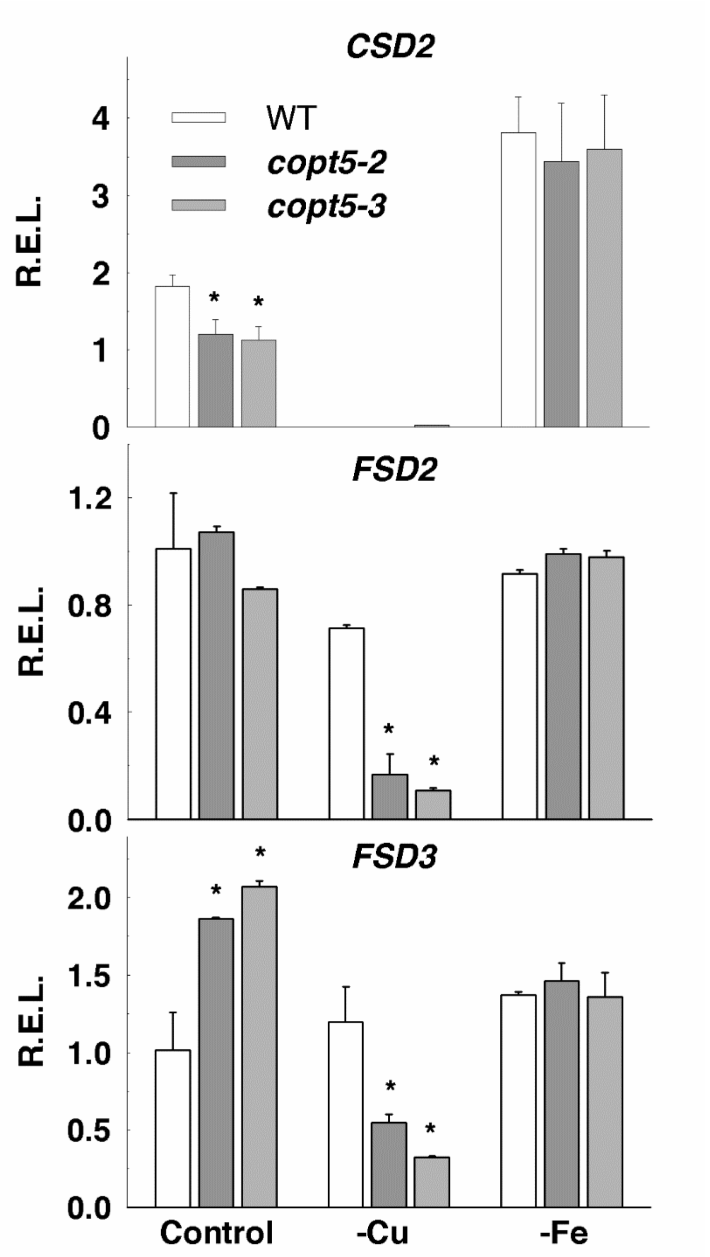

Fig. S6 *SOD* expression in the WT and *copt5* seedlings. *CSD2, FSD2* and *FSD3* relative expression levels (R.E.L.) in the *copt5* mutant. The qRT-PCR analysis in the 7-day-old WT, *copt5-2* and *copt5-3* seedlings grown under the same conditions used in Fig. 6. Bars correspond to arithmetic means ($2^{-\Delta\Delta Ct}$) ± SD (n=3). * indicates statistical differences ($P < 0.05$) between the values of the the WT and *copt5* mutants in each condition.

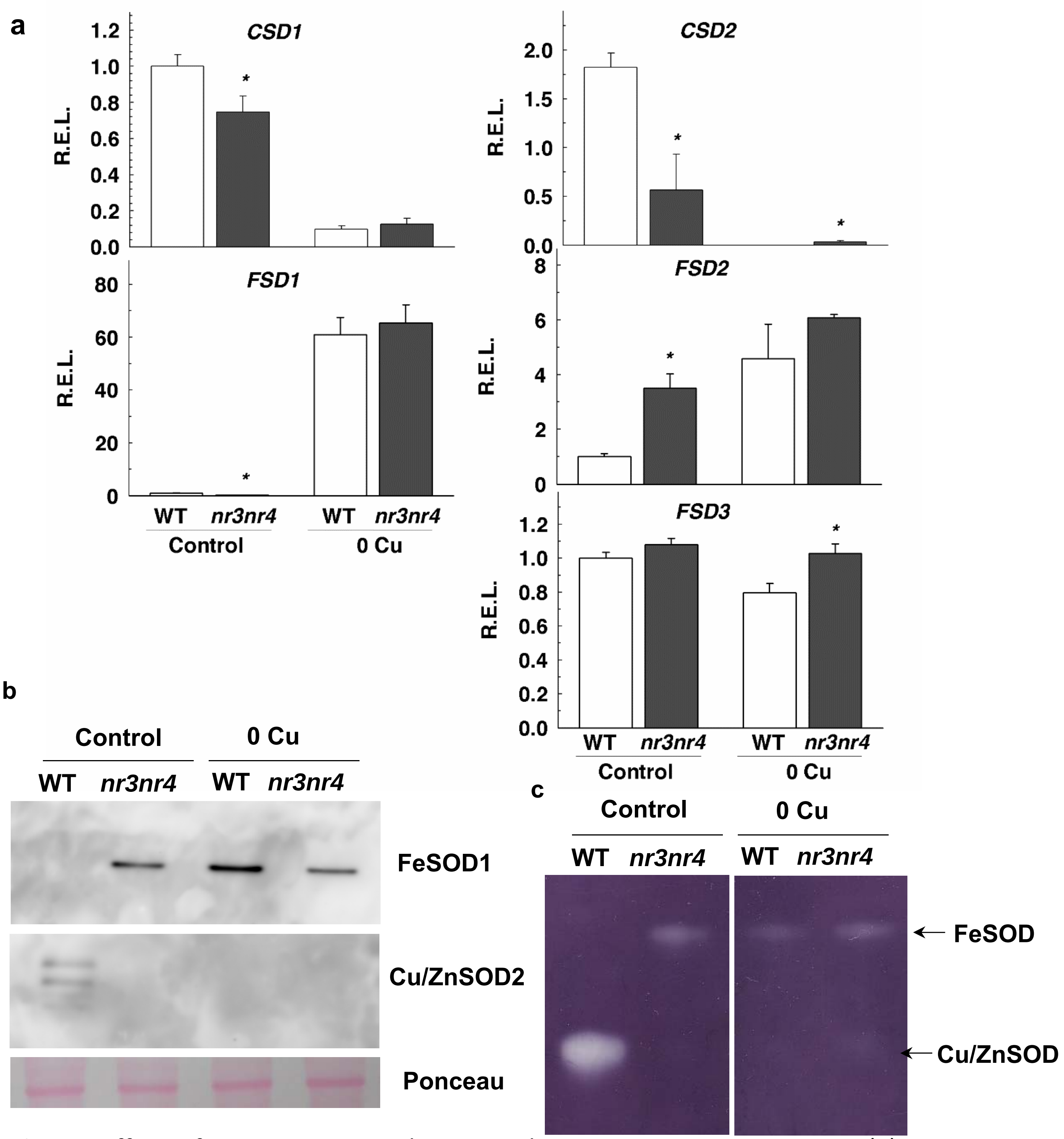

Fig. S7 Effect of Cu on SOD regulation in the *nramp3nramp4* mutant. (a) *FSD1, CSD1, CSD2, FSD2* and *FSD3* relative expression levels (R.E.L.). The qRT-PCR analysis in the 7-day-old WT and *nramp3nramp4* (*nr3nr4*) seedlings grown under the same conditions used in Fig. 5c. Bars correspond to arithmetic means ($2^{-\Delta\Delta Ct}$) ± SD (n=3). * indicates statistical differences (P < 0.05) between the values of the the WT and *copt5* mutants in each condition. (b) Immuno-detection of FSD1 and CSD2 using 35 µg of protein extract. Ponceau staining is shown as a loading control. (c) The SOD enzyme activities analysed in native gels loaded with 100 µg of protein extract. The gel was stained for total SOD activity. Full-length blots/gels and replicates are presented in Supplementary Figure S10.

a

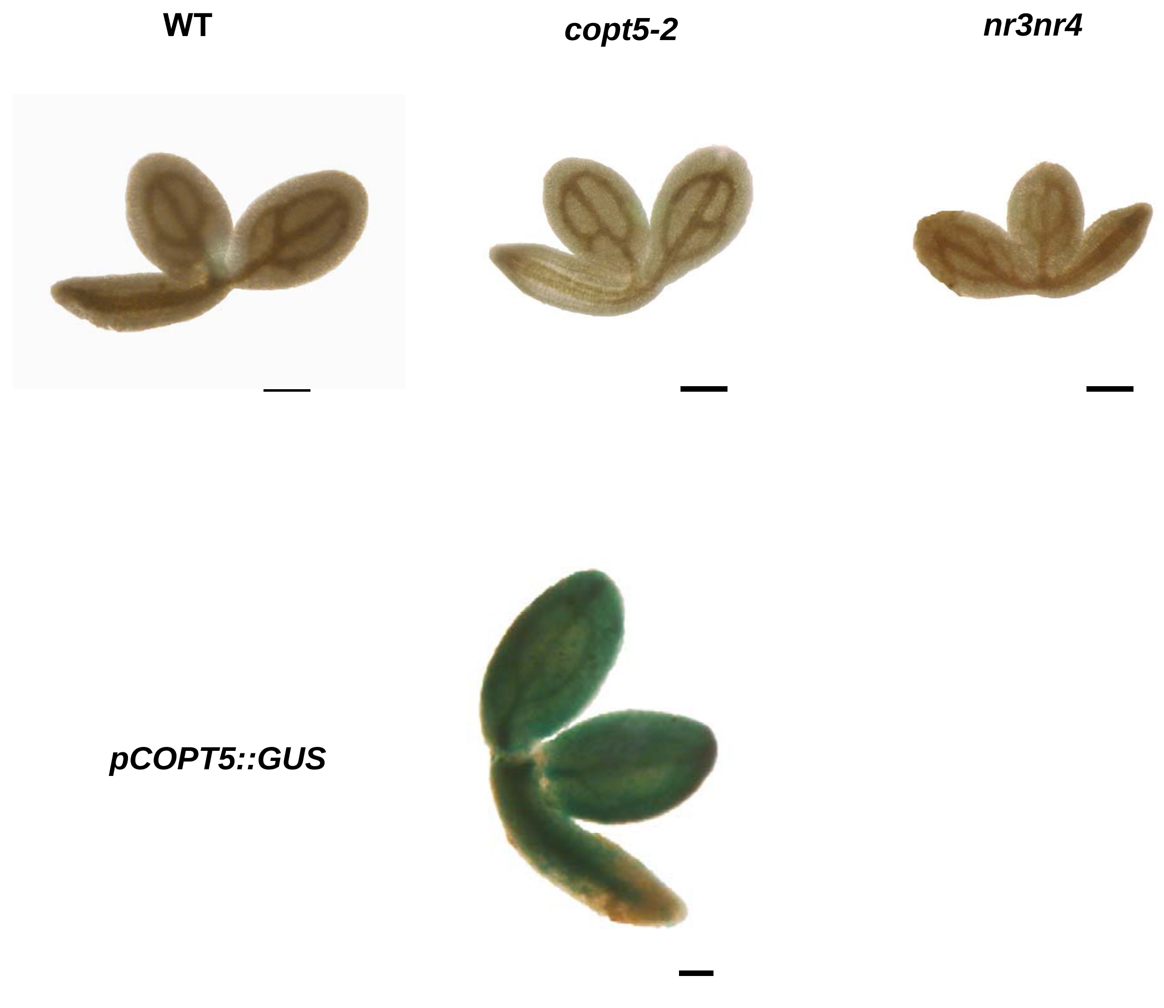

Fig. S8 Fe localization and *COPT5* expression in dry seed embryos. (a) Perls/DAB staining of the dry seed embryos from the WT, *copt5-2* and *nramp3nramp4* mutants (b) *COPT5* expression in embryos. GUS expression in a representative *pCOPT5::GUS* dry seed embryo.

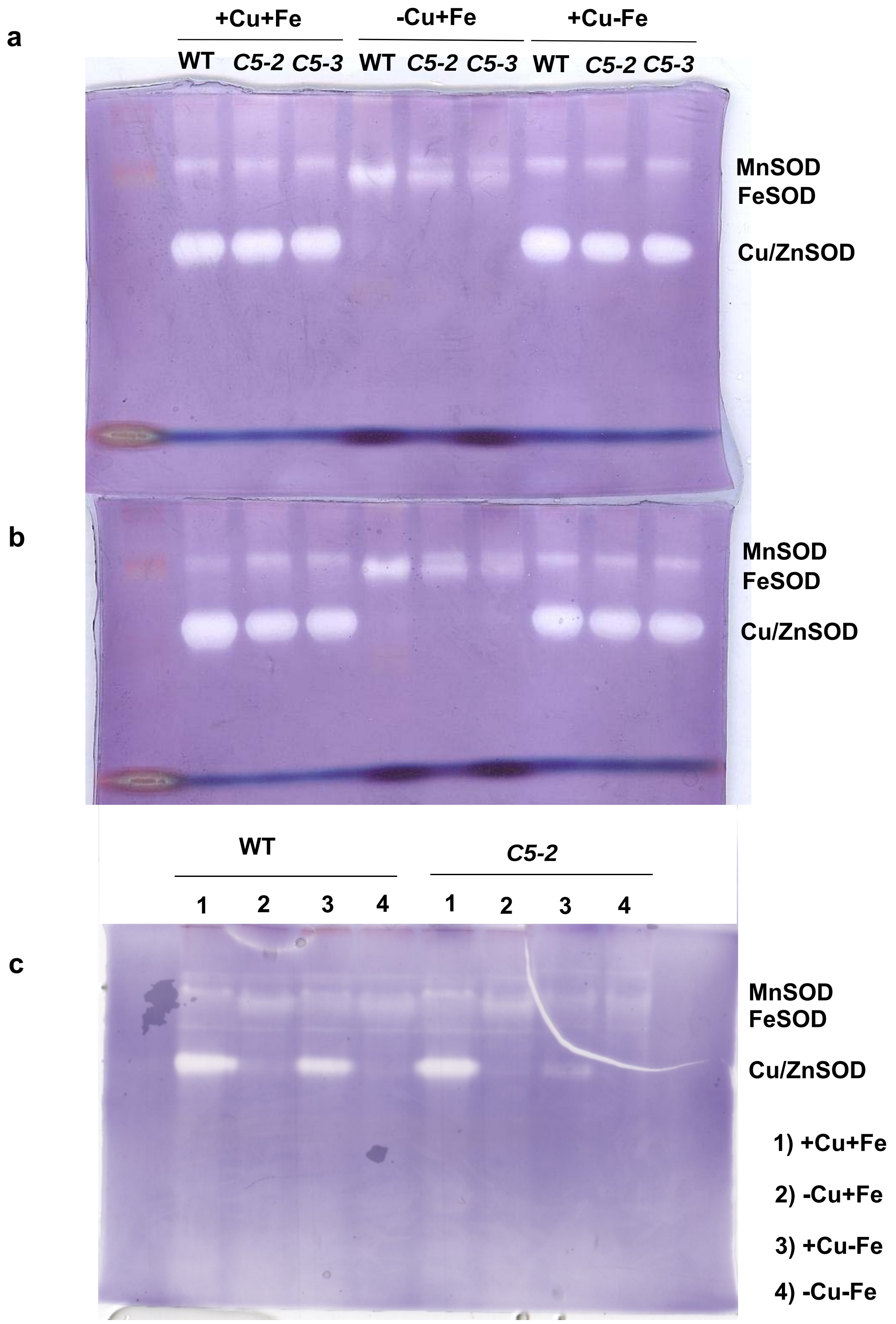

Fig. S9 Effect of Cu availability on SOD regulation in the *copt5* mutants. SOD enzyme activities analysed in native gels loaded with 100 µg of protein extract. The gels were stained for total SOD activity. Replicate blots are shown. a) is the whole blot shown in Fig 6.

d

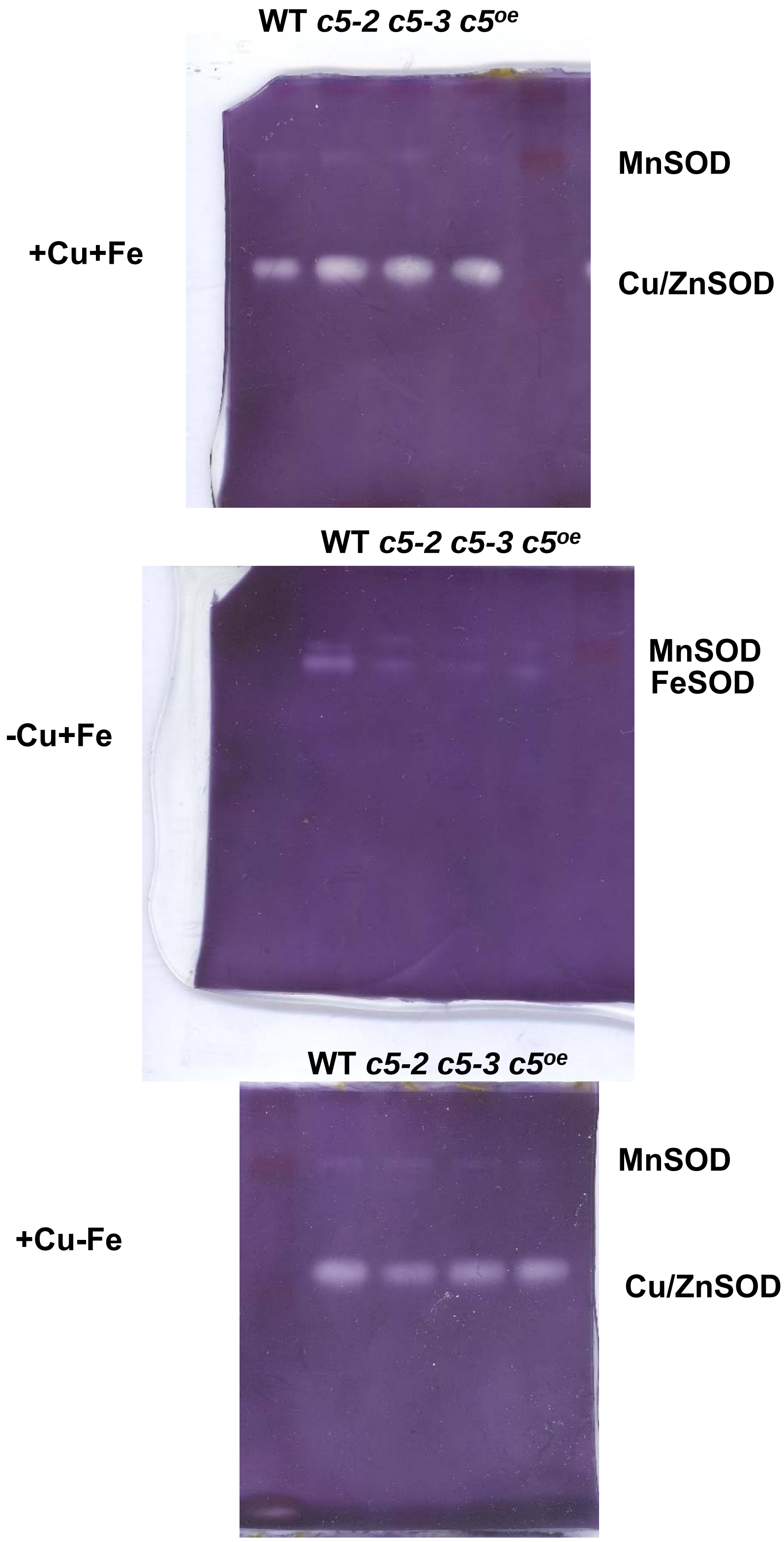

Fig. S9 (continuation) Effect of Cu availability on SOD regulation in the *copt5* mutants. SOD enzyme activities analysed in native gels loaded with 100 µg of protein extract. The gels were stained for total SOD activity. Top gel is the whole blot shown in Fig 6 and replicate blots are shown.

e

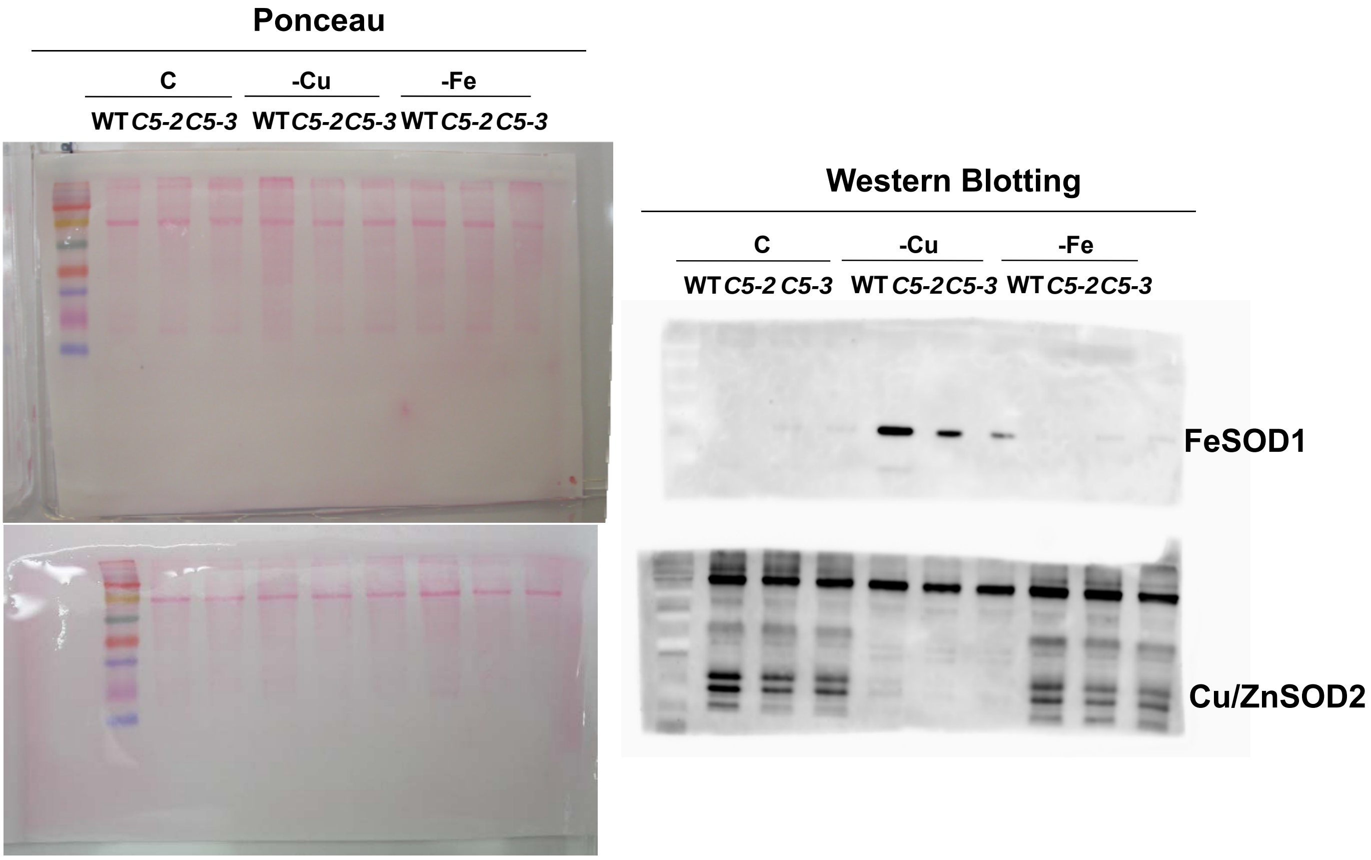

Fig. S9 (continuation). Effect of Cu availability on SOD regulation in the *copt5* mutants. Ponceau staining is shown as a loading control (left). Immuno-detection of FSD1 and CSD2 using 35 µg of protein extract (right). Whole blots from Fig 6 panel b.

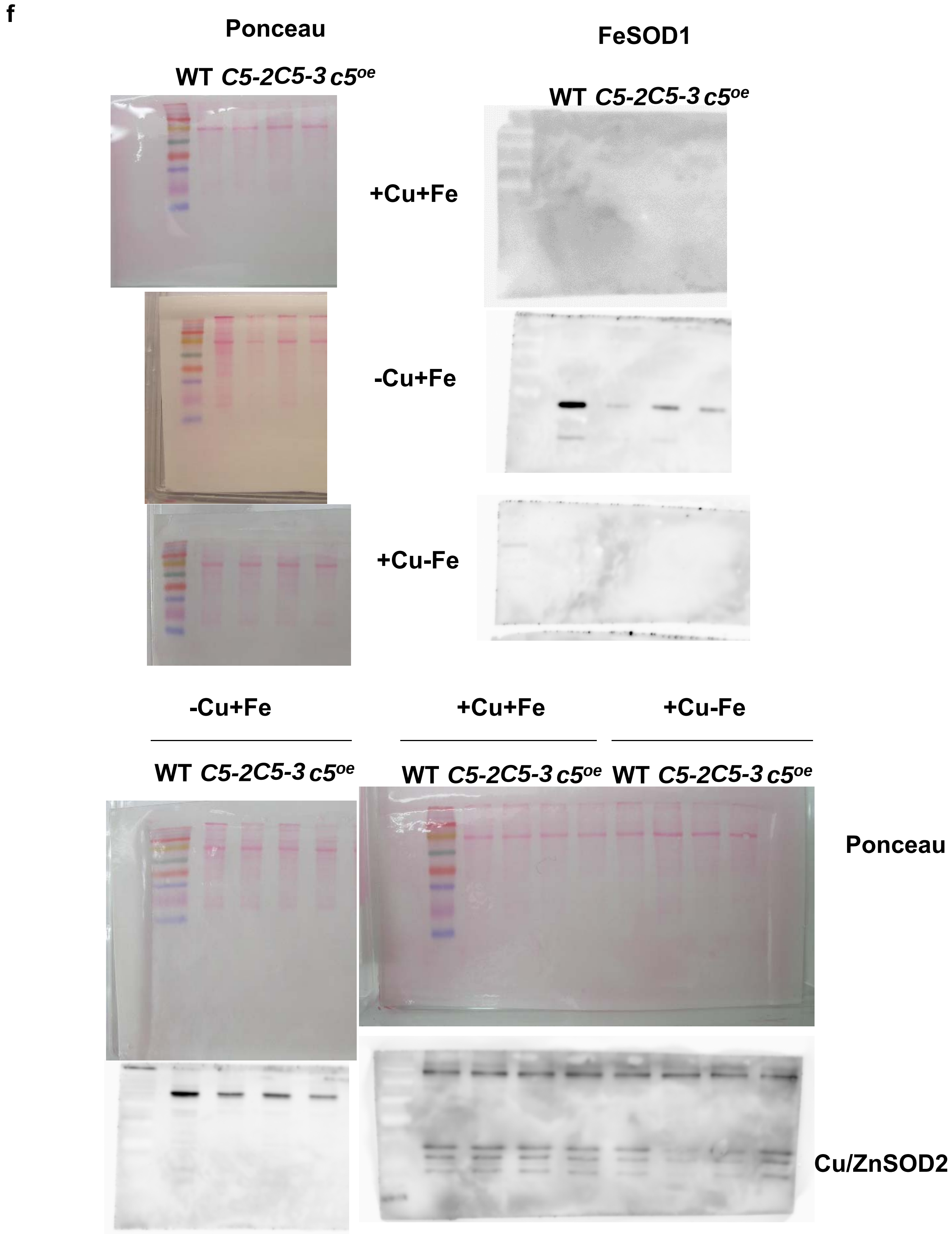

Fig. S9 (continuation) Effect of Cu availability on SOD regulation in the *copt5* mutants. Immuno-detection of FSD1 and CSD2 using 35 µg of protein extract. Ponceau staining is shown as a loading control. Replicates from Fig 6 panel b blots are shown.

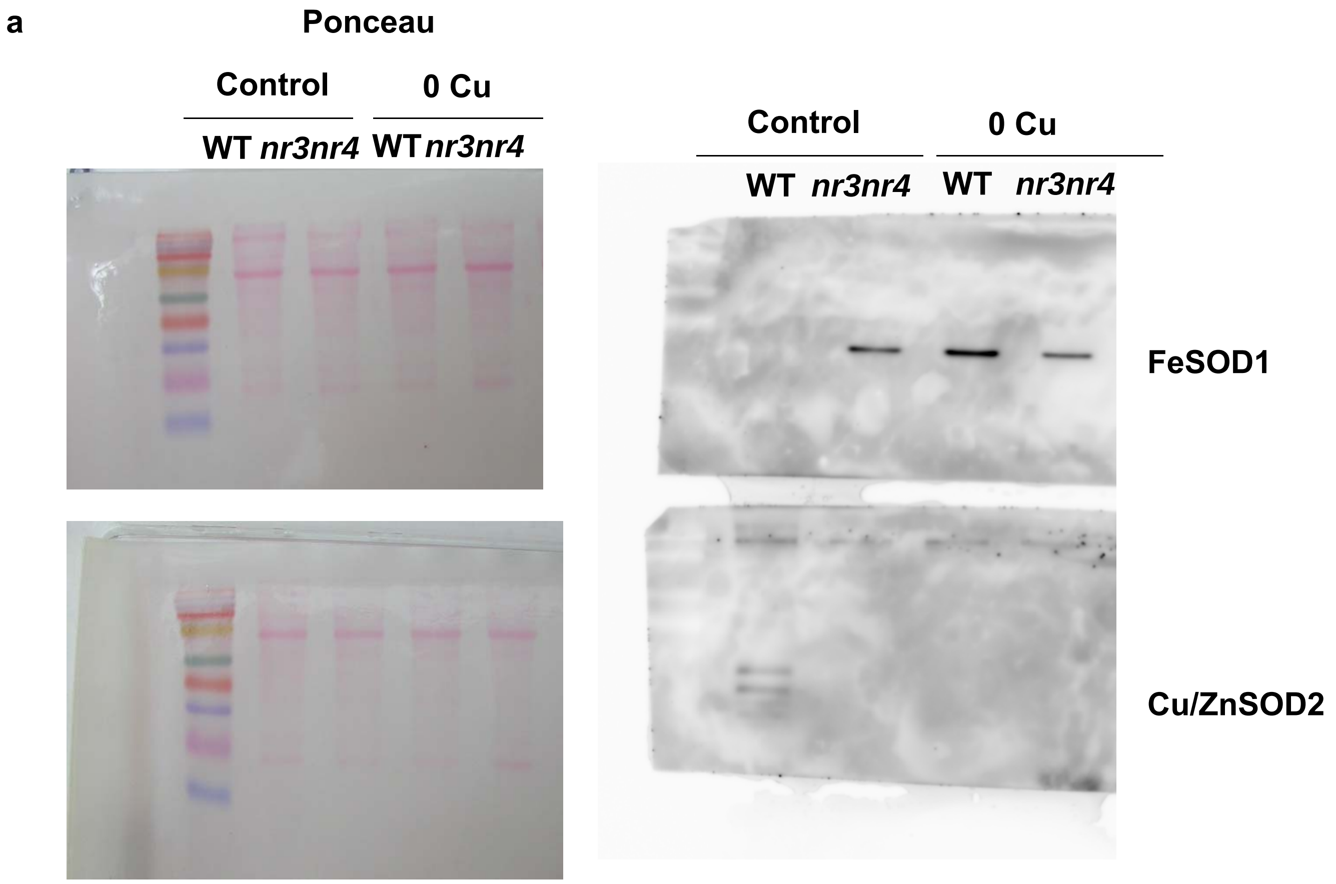

Fig. S10 Effect of Cu on SOD regulation in the *nramp3nramp4* mutant. Immuno-detection of FeSOD1 and Cu/ZnSOD2 using 35 µg of protein extract. Ponceau staining is shown as a loading control. Whole blots shown Supplemental Figure S7 in panel b.

b

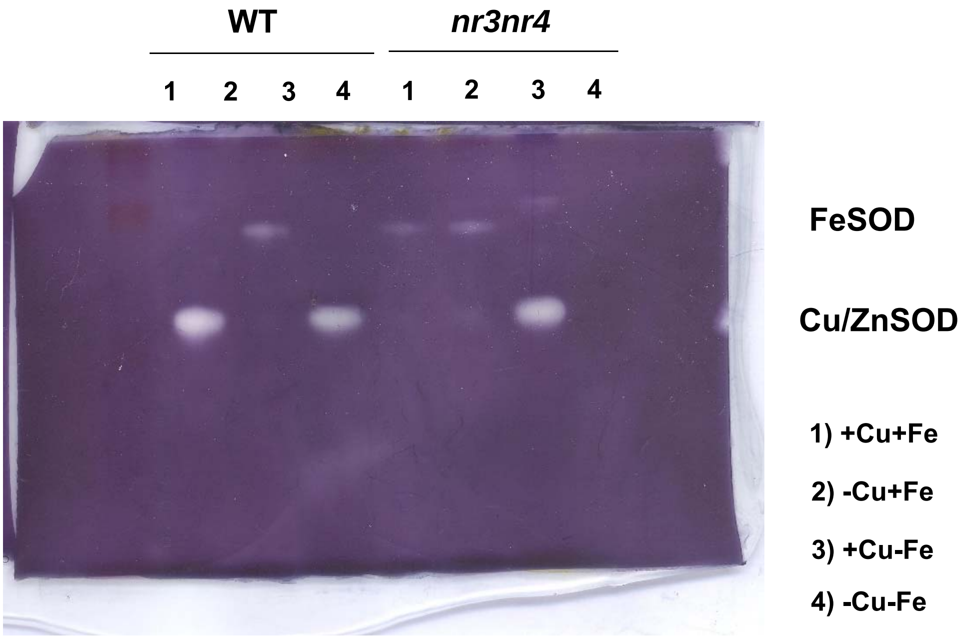

c

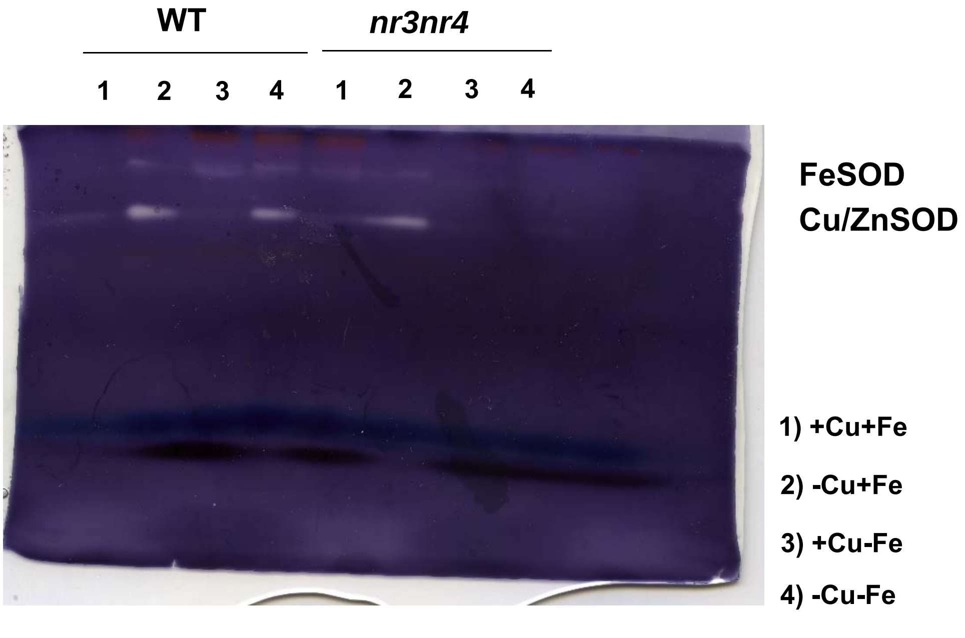

Fig. S10 (continuation) Effect of Cu on SOD regulation in the *nramp3nramp4* mutant. The SOD enzyme activities analysed in the native gels loaded with 100 µg of protein extract. The replicate gels were stained for total SOD activity. a) is the whole blot shown in Supplemental Figure S7 panel c.

Table S1. Primers used for the qRT-PCR analysis. The r^2 values of the regression curves for each individual gene by comparing microarrays. The qPCR expression data are indicated.

GENE	FORWARD (5'→3')	REVERSE (5'→3')	r^2 value
BHLH38	AGAGCTGCAACAGCAAGTGA	ACCAAGCCTAGTGGCAGAAA	
BHLH39	CAGAGCTGCAAGAGCAAGTG	ACCAAGCCTAGTCGCAGAAA	
BHLH100	AAACCGACGACGTATCCAAC	GATTGGTGGGAGGAGACAA	
BHLH101	TTGCTGTCCAGTTGCTACG	GGCGTAATCCCAAGAGACATA	
BRUTUS	GCTCTGGCACAAGTCAATCA	CGTTCATCAAATGCCGATAA	
COPT2	CCTTTCGTATTTGGTGATGCT	AAACACCTGCGTTAAAGGAC	0.92
COPT5	TTGCAGCTATGTCTTTCAA	CGGCGGTTAATCCGACGACA	0.8
CSD1	CATCATTGGTCTCCAGGGCT	GACCTCCTTATTACATCAAT	0.89
CSD2	GTCCTACAACTGTGAAT	TCCATGAGGCCCTGGAGT	0.81
FIT	TTTTCGCGGTATCAATCCTC	GGTATGTGTCCGGAGAAGGA	
FRO2	GTTGGTTTATAGCCCGACGA	GGGCCGTAAGGACCTTCTAC	
FRO3	GATTCTACTGGCTTCTCTTGG	CTAATCCGGCCTTCACTAAC	
FSD1	ACCGAAGACCAGATTACATA	TGGCACTTACAGCTTCCCAA	0.98
FSD2	CACAGCTGGATTTGAGCTGA	AATCTGAGGAGCTCTCCAGT	0.95
FSD3	GGTCTCGAGGTGGTTTAAAG	AACACCCTTTTGAGGCGTGT	0.91
IRT1	CCCCGCAAATGATGTTACCTT	GGTATCGCAAGAGCTGTGCAT	
NRAMP3	ATGGTTTTGTGGGTTATGGC	CTGAGCTTCCTTATTCCGT	
NRAMP4	TTGGAGCATTGGTCCCTAAG	GAAAGAAACCGCAAGAGCAC	
UBQ10	TAATCCCTGATGAATAAGTGTTCTAC	AAAACGAAGCGATGATAAAGAAG	
YSL1	ACAAGGAGATGCACAGGCCAAGAAA	TCACAGCCGCGATGACAAAAAGAC	
YSL3	ATTGGCCAGGAAACAAGTGTTTGGGT	GACAAGTCCCGCGACTACACCATTT	

Table S2. Biological processes overrepresented in the global GO analysis (FatiGo, P<0.05). The differentially expressed genes were identified by applying a false discovery rate (FDR) lower than 1% and a 1.5-fold change ($\log_2 |1.5|$).

	Comparison			
Biological Process	C5 Cu vs WT Cu	C5 BCS vs WT BCS	WT BCS vs WT Cu	C5 BCS vs C5 Cu
**primary metabolic process**				
**carbohydrate biosynthetic process (GO:0016051)**		↓		↓
cellular carbohydrate metabolic process (GO:0044262)		↓		↓
polysaccharide metabolic process (GO:0005976)			↓	↓
trehalose biosynthetic process (GO:0005992)			↓	
cellular glucan metabolic process (GO:0006073)			↓	↓
**lipid metabolic process (GO:0006629)**		↓		
lipid catabolic process (GO:0016042)		↓		
fatty acid metabolic process (GO:0006631)	↓	↓		
fatty acid biosynthetic process (GO:0006633)		↓		
**secondary metabolic process**				
**sulfur metabolic process (GO:0006790)**		↓		↓
**glucosinolate metabolic process (GO:0019760)**		↓		↓
**toxin catabolic process**			↑	↑
response to toxin (GO:0009636)			↑	↑
indole phytoalexin biosynthetic process (GO:0009700)			↓	
camalexin biosynthetic process (GO:0010120)			↓	↓
**lignin catabolic process (GO:0046274)**			↓	↓
**response to external stimulus**				
**response to biotic stimulus (GO:0009607)**				
defense response to fungus (GO:0050832)			↓	↓
response to fungus (GO:0009620)			↓	↓
response to chitin (GO:0010200)			↓	↓
immune response (GO:0006955			↓	↓
innate immune response (GO:0045087)			↓	↓
cell death (GO:0008219)			↓	↓
apoptosis (GO:0006915)			↓	↓
programmed cell death (GO:0012501)			↓	↓
host programmed cell death induced by symbiont (GO:0034050)			↓	
plant-type hypersensitive response (GO:0009626)			↓	
defense response, incompatible interaction (GO:0009814)			↓	↓
defense response to bacterium, incompatible interaction (GO:0009816)			↓	↓
**response to abiotic stimulus**				
response to osmotic stress (GO:0006970)			↓	
response to radiation (GO:0009314)			↑	↑
response to light stimulus (GO:0009416)			↑	↑
response to red or far red light (GO:0009639)				↑
response to red light (GO:0010114)			↑	↑
response to absence of light (GO:0009646)			↓	↓
response to mechanical stimulus (GO:0009612)			↓	↓
**response to endogenous stimulus**				
**response to reactive oxygen species (GO:0000302)**		↑	↑	↑
oxygen and reactive oxygen species metabolic process (GO:0006800)		↑	↑	↑
response to oxidative stress (GO:0006979)		↑	↑	↑
response to hydrogen peroxide (GO:0042542)		↑	↑	↑
hydrogen peroxide catabolic process (GO:0042744)		↑	↑	↑
**response to carbohydrate stimulus (GO:0009743)**			↓	↓
**response to hormone stimulus (GO:0009725)**			↓	↓
response to auxin (GO:0009733)			↓	↓
response to abscisic acid (GO:0009737)			↓	↓
response to gibberellin (GO:0009739)			↓	↓
response to brassinosteroid (GO:0009741)			↓	↓
response to salicylic acid (GO:0009751)			↓	↓
response to jasmonic acid (GO:0009753)			↓	↓
response to ethylene (GO:0009723)		↑	↓	↓
hormone-mediated signaling pathway (GO:0009755)		↑	↓	↓
intracellular signaling cascade (GO:0007242)		↑	↓	↓
two-component signal transduction system (phosphorelay) (GO:0000160)		↑	↓	↓
ethylene-activated signaling pathway (GO:0009873)		↑	↓	↓
regulation of ethylene-activated signaling pathway (GO:0010104)		↑		
negative regulation of signal transduction (GO:0009968)		↑		
**cellular homeostasis**				
**peptide transport (GO:0015833)**		↑		↑
oligopeptide transport (GO:0006857)		↑		↑
**cellular ion homeostasis (GO:0006873)**		↑		↑
transition metal ion transport (GO:0000041)		↑		↑
iron ion transport (GO:0006826)		↑		↑
